# The mechanism of the triple aryne–tetrazine reaction cascade: theory and experiment†
†Electronic supplementary information (ESI) available: Experimental protocols, characterization data, and NMR spectra of all new compounds. See DOI: 10.1039/c8sc01796d


**DOI:** 10.1039/c8sc01796d

**Published:** 2018-08-23

**Authors:** Sung-Eun Suh, Shuming Chen, K. N. Houk, David M. Chenoweth

**Affiliations:** a Department of Chemistry , University of Pennsylvania , 231 South 34th Street , Philadelphia , Pennsylvania 19104 , USA . Email: dcheno@sas.upenn.edu; b Department of Chemistry and Biochemistry , University of California , Los Angeles , California 90095-1569 , USA

## Abstract

This article describes an experimental and computational investigation on the possible aryne reactivity modes in the course of the reaction of two highly energetic molecules, an aryne and a 1,2,4,5-tetrazine. Beyond the triple aryne–tetrazine (TAT) reaction, it was observed that combinations of several reactivity modes afford several heterocyclic compounds. Density Functional Theory (DFT) calculations of competition between a second Diels–Alder reaction and the nucleophilic addition pathways indicates the latter to be more favorable. Crossover experiments and computational study of the proton transfer step reveal that the reaction proceeds intermolecularly with the assistance of a water molecule, rather than intramolecularly. The resulting enamine intermediate was found to undergo either a stepwise formal [2 + 2] or [4 + 2] cycloaddition, and their energetic profiles were compared against each other. Isolation of an ene-product and a rearranged product shows the potential competition with oxidation/desaturation. These studies show how multiple arynes react with a highly reactive starting material and provide guidance for future applications of aryne-based multicomponent cascade reactions.

## Abstract 2

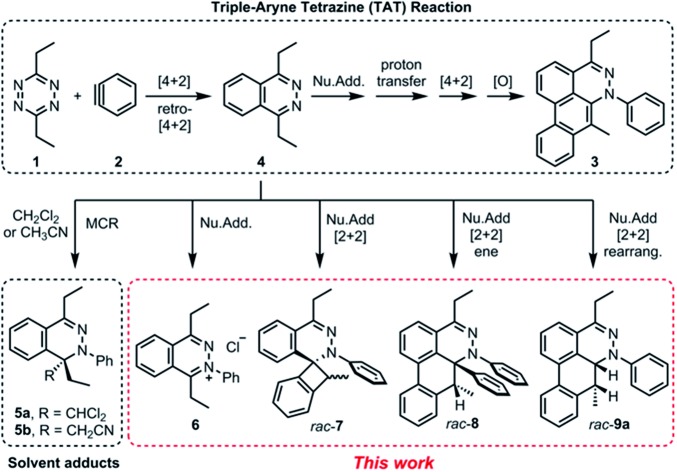
This article describes an experimental and computational investigation on the possible aryne reactivity modes in the course of the reaction of two highly energetic molecules, an aryne and a 1,2,4,5-tetrazine.

## Introduction

**Fig. 1:**
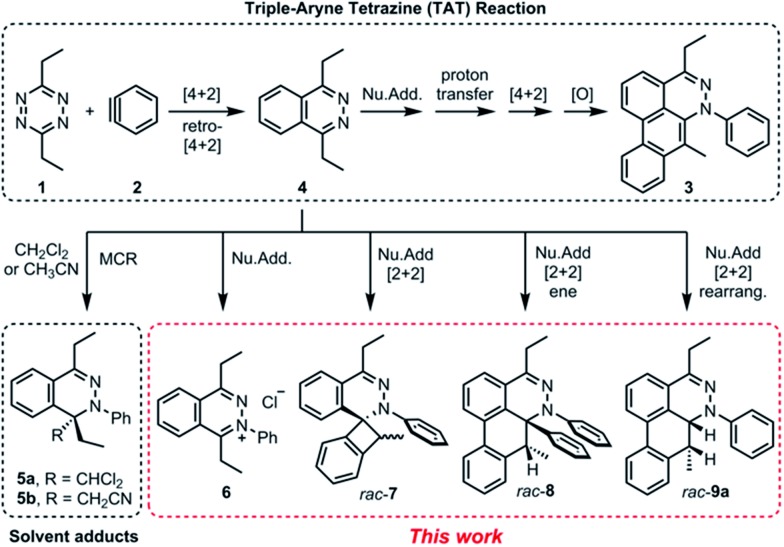
Reactivity modes of 1,2,4,5-tetrazine and arynes. [4 + 2], [4 + 2] cycloaddition; Nu.Add, nucleophilic addition; [O], oxidation; MCR, multicomponent reaction; ene, ene-reaction; rearrang., rearrangement.

Recently, we reported a new multistep process termed the triple aryne–tetrazine (TAT) reaction.^1^ The reaction combined diverse reactivity modes between simple 1,2,4,5-tetrazine **1** (henceforth referred to as “tetrazine”) starting materials^2^ and aryne **2** into a single multistep process, resulting in the addition of three aryne equivalents to a tetrazine core. In the TAT reaction, dibenzocinnoline **3** and the cycloaddition intermediate **4** were isolated. In addition, we reported solvent participation in aryne reactions with the two solvent-trapped intermediates **5a** and **5b** (Fig. 1).^3^ Detailed investigation of these side reactions can yield valuable insight into aryne chemistry^4^ and provide access to new heterocyclic scaffolds.^5^ Here we report new mechanistic aspects of the reaction between a tetrazines and arynes, elucidating unique divergent pathways to alternative products that arise during the reaction course. The reaction mechanism consists of six consecutive elementary steps. As many as five steps can directly engage the aryne.^6^ Using a combination of experimental and computational methods, we elucidate the unique competing pathways that are operational throughout the course of the reaction to yield products **6**, *rac*-**7**, *rac*-**8**, and *rac*-**9a**.

## Results and discussion

**Fig. 2:**
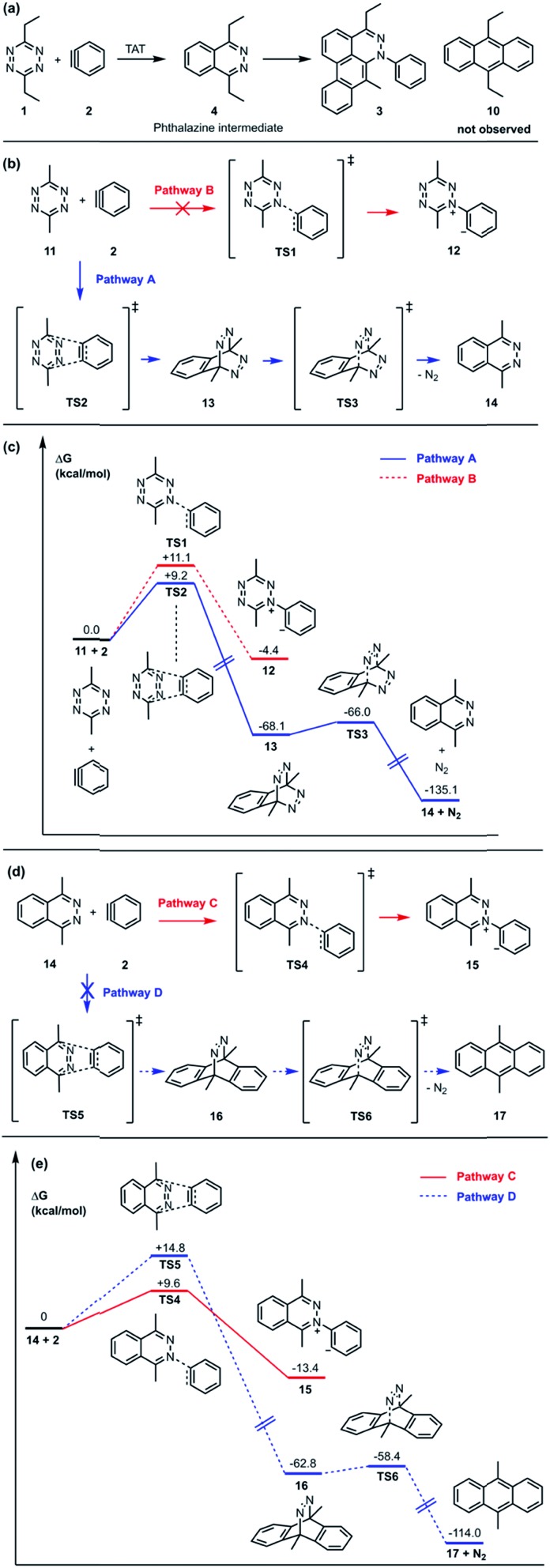
(a) The reported TAT reaction and isolated phthalazine intermediate **4**. (b) Diels–Alder reaction (pathway A) *vs.* nucleophilic addition (pathway B) from tetrazine **11** and aryne **2**. (c) Free energy profiles of pathways A and B proceeding from tetrazine **11** and aryne **2** computed at the M06-2X/6-311+G(d,p)/SMD (CH_2_Cl_2_)//M06-2X/6-31G(d) level of theory. (d) Nucleophilic addition (pathway C) *vs.* Diels–Alder reaction (pathway D) from phtalazine **14** and aryne **2**. (e) Free energy profiles of pathways C and D proceeding from phthalazine **14** and aryne **2** computed at the M06-2X/6-311+G(d,p)/SMD (CH_2_Cl_2_)//M06-2X/6-31G(d) level of theory. Gibbs free energies are reported in kcal mol^–1^.

**Fig. 3:**
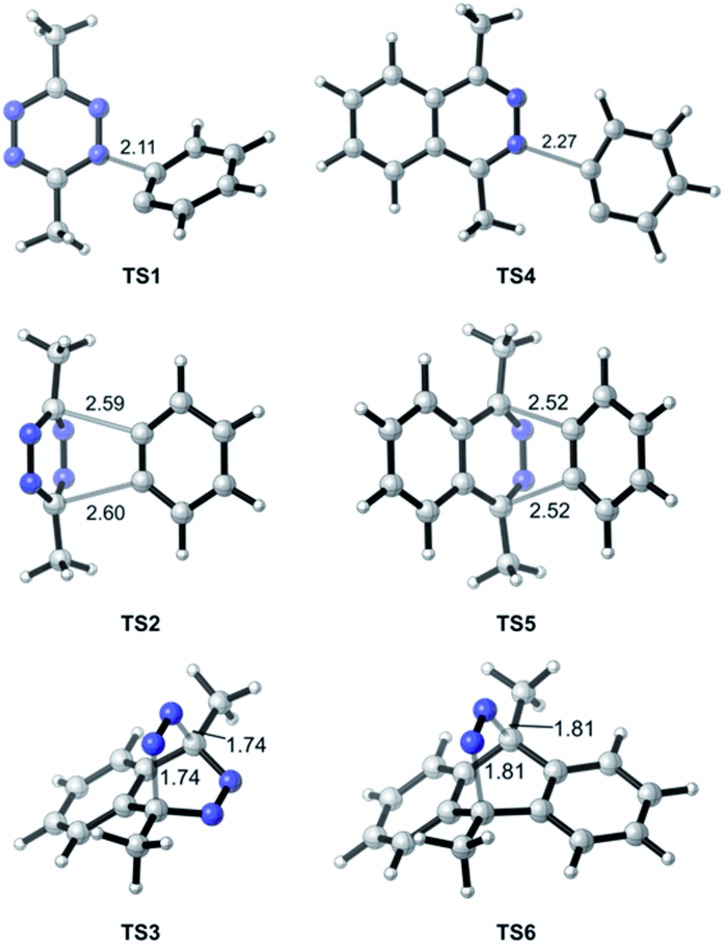
Optimized geometries of transition states along pathways A–D. Interatomic distances are shown in ångströms.

In the TAT reaction, it was observed that **1** undergoes [4 + 2] cycloaddition with **2** and loss of N_2_ affords the phthalazine intermediate **4**^7^ followed by several further mechanistic steps to give dibenzocinnoline **3** (Fig. 2A). In the TAT reaction, anthracene **10** was not observed,^8^ indicating that the phthalazine intermediate **4** does not undergo a further [4 + 2] cycloaddition. Instead, **4** is observed to undergo a nucleophilic addition to another equivalent of **2**. We used Density Functional Theory (DFT) calculations to compare free energy profiles of the Diels–Alder *versus* the nucleophilic addition reaction pathways for both the tetrazine and benzyne pair and the phthalazine and benzyne pair. To simplify the calculations, the ethyl substituents on tetrazine **1** were modeled with methyl groups (Fig. 2A and B). Two pathways are possible: nucleophilic addition of tetrazine **11** with aryne **2** 9 or a Diels–Alder cycloaddition. The two possible pathways were evaluated using DFT and the free energy profile for the reactions are shown in Fig. 2C. The Diels–Alder transition state **TS2** is lower in energy than the nucleophilic addition transition state **TS1** by 1.9 kcal mol^–1^ (Fig. 2B, C and 3). For phthalazine **14** and aryne **2**, the nucleophilic addition is more favorable by 5.2 kcal mol^–1^ (Fig. 2D, E, and 3). These results are in good agreement with the experimental observation that the first Diels–Alder reaction is favored over the nucleophilic addition pathway, but the second Diels–Alder reaction is outcompeted by nucleophilic addition. Both nucleophilic addition pathways are overall somewhat exergonic, whereas both Diels–Alder pathways are strongly exergonic, with very low barriers for dinitrogen extrusion from the Diels–Alder adducts.^10^,^11^

**Scheme 1:**
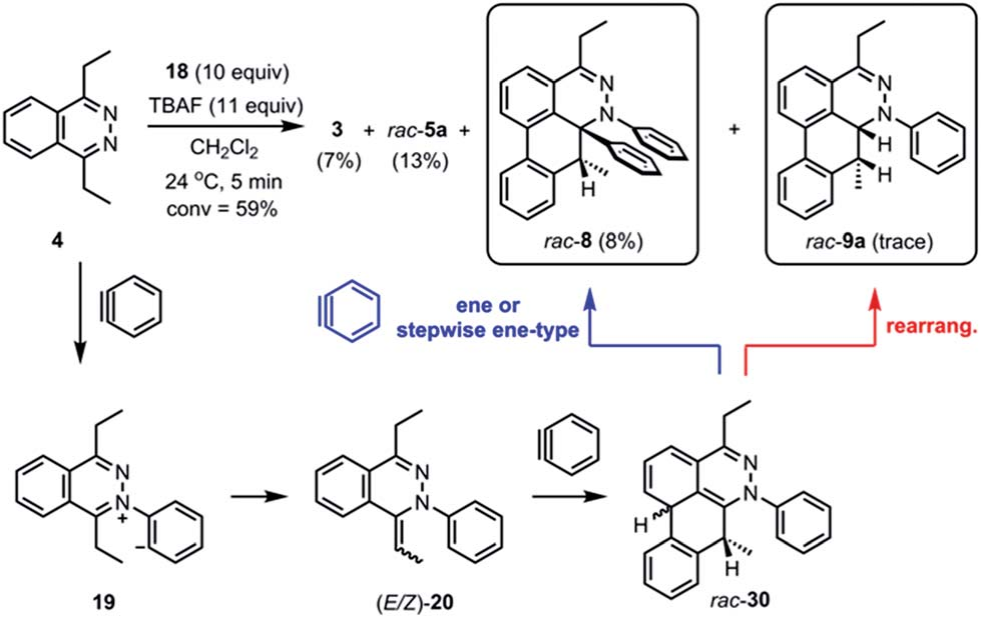
Ene or stepwise ene-type reaction and rearrangement of the intermediate *rac*-**30**.

**Fig. 4:**
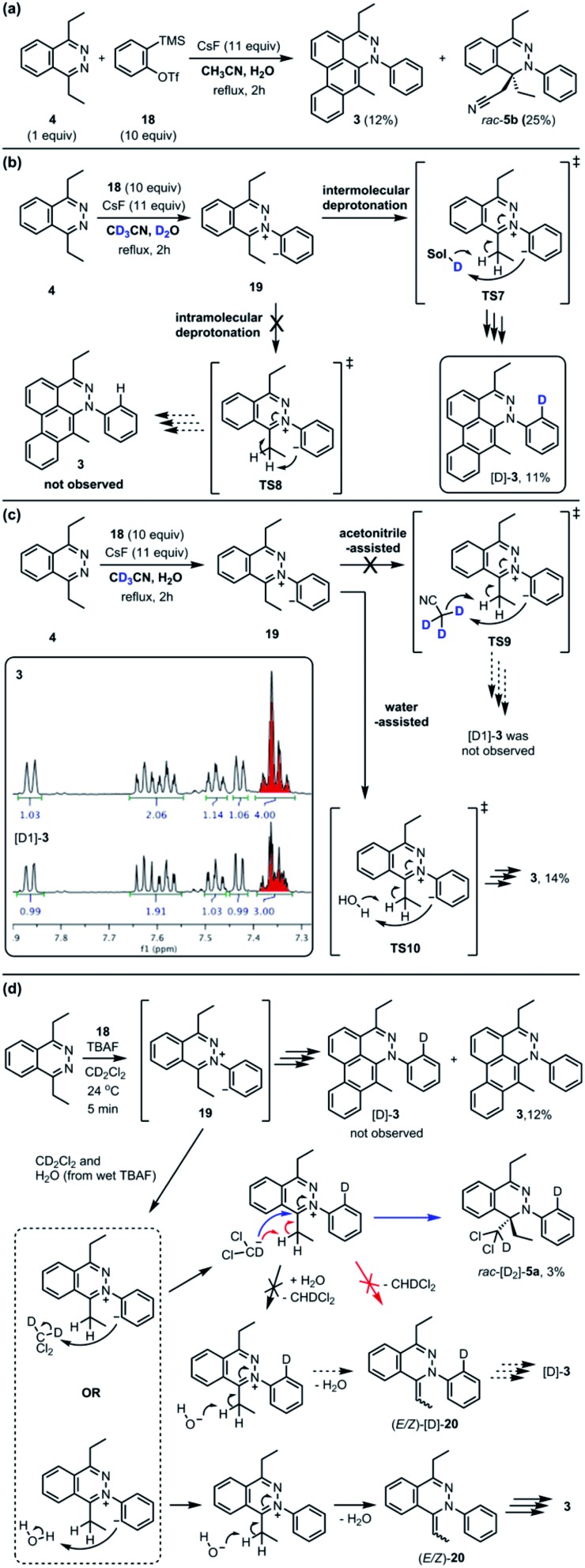
Crossover experiment to determine intra-, acetonitrile-assisted inter-, or water-assisted intermolecular proton transfer. (a) The reaction was conducted in the mixture of 1 : 1 ratio of CH_3_CN/H_2_O. (b) The mixture of 1 : 1 ratio of CD_3_CN/D_2_O. (c) The mixture of 1 : 1 ratio of CD_3_CN/H_2_O. (d) Importance of water in the previous crossover experiment.^3^

The standard TAT reaction is conducted with **1**, 11 equivalents of tetrabutylammonium fluoride (TBAF) in THF, and 10 equivalents of 2-(trimethylsilyl)phenyl trifluoromethanesulfonate **18** in CH_2_Cl_2_ at 24 °C, to produce product **3** in 44% yield within 5 minutes.^1^ The intermediate **4** is isolable from the TAT reaction when conducted with an excess of tetrazine **1**. The reaction between **18** and the intermediate **4** under the standard TAT reaction condition afforded the desired product **3** and *rac*-**5b** in 7% and 13% yield, respectively (Scheme 1).^3^ Here, using anhydrous CsF instead of TBAF, which has a low concentration of water and is considered a wet fluoride source, we conducted the reaction in CH_3_CN under reflux. Higher conversion and higher yield of both **3** and *rac*-**5b** were observed (Fig. 4A). The source of low mass balance for the TAT sequence is due to competing aryne and tetrazine decomposition pathways under the reaction conditions. To discriminate between an intra- *versus* intermolecular proton transfer pathway, the reaction was performed in CD_3_CN and D_2_O under reflux (Fig. 4B). Only deuterated dibenzo[*de*,*g*]cinnoline [D]-**3** was isolated (11% yield), as analyzed by ^1^H NMR experiment. The result indicates that the intermediate **19** most likely did not proceed through an intramolecular pathway but instead through an intermolecular proton transfer pathway, assisted by a fluoride, a hydroxide, conjugate base of CH_3_CN, or a phenyl anion of another benzyne adduct. To further probe solvent participation in the proton transfer step, experiments were conducted in CD_3_CN and H_2_O (Fig. 4C). According to the result shown in Fig. 4B, the intramolecular pathway is unlikely to be favored. Two possibilities arise for the intermolecular pathway, a water-mediated or CH_3_CN-mediated proton transfer, assuming H_2_O can only serve as the proton source and CD_3_CN can only provide deuterium during the proton transfer step. After this reaction, only non-deuterated **3** was observed in 14% yield, which ruled out proton transfer with the conjugate base of CH_3_CN, supporting a water-mediated proton transfer pathway. These results account for why only **3** and *rac*-[D_2_]-**5a** were found and [D]-**3** was not observed in our previous crossover experiments performed in CD_2_Cl_2_ (Fig. 4D).^3^ When it comes to the stepwise mechanism in Fig. 4D, after deuterium transfer from CD_2_Cl_2_ to the intermediate **19**, the deuterated cationic intermediate is formed. According to the isolation of **3** and *rac*-[D_2_]-**5a**, the cationic intermediate may only react with dichloromethanide-d *via* 1,2-addition to provide *rac*-[D_2_]-**5a** but may not undergo further deprotonation assisted by either dichloromethanide-d or hydroxide to afford (*E*/*Z*)-[D]-**20** because [D]-**3** was not detected. A plausible pathway to account for formation of **3** may involve a water-mediated proton transfer to give (*E*/*Z*)-**20**. As shown above, water is an important proton source for the TAT reaction, and this result is consistent with the observation that no desired product from the TAT reaction is observed with anhydrous tetrabutylammonium difluorotriphenylsilicate (TBAT).^1^

**Fig. 5:**
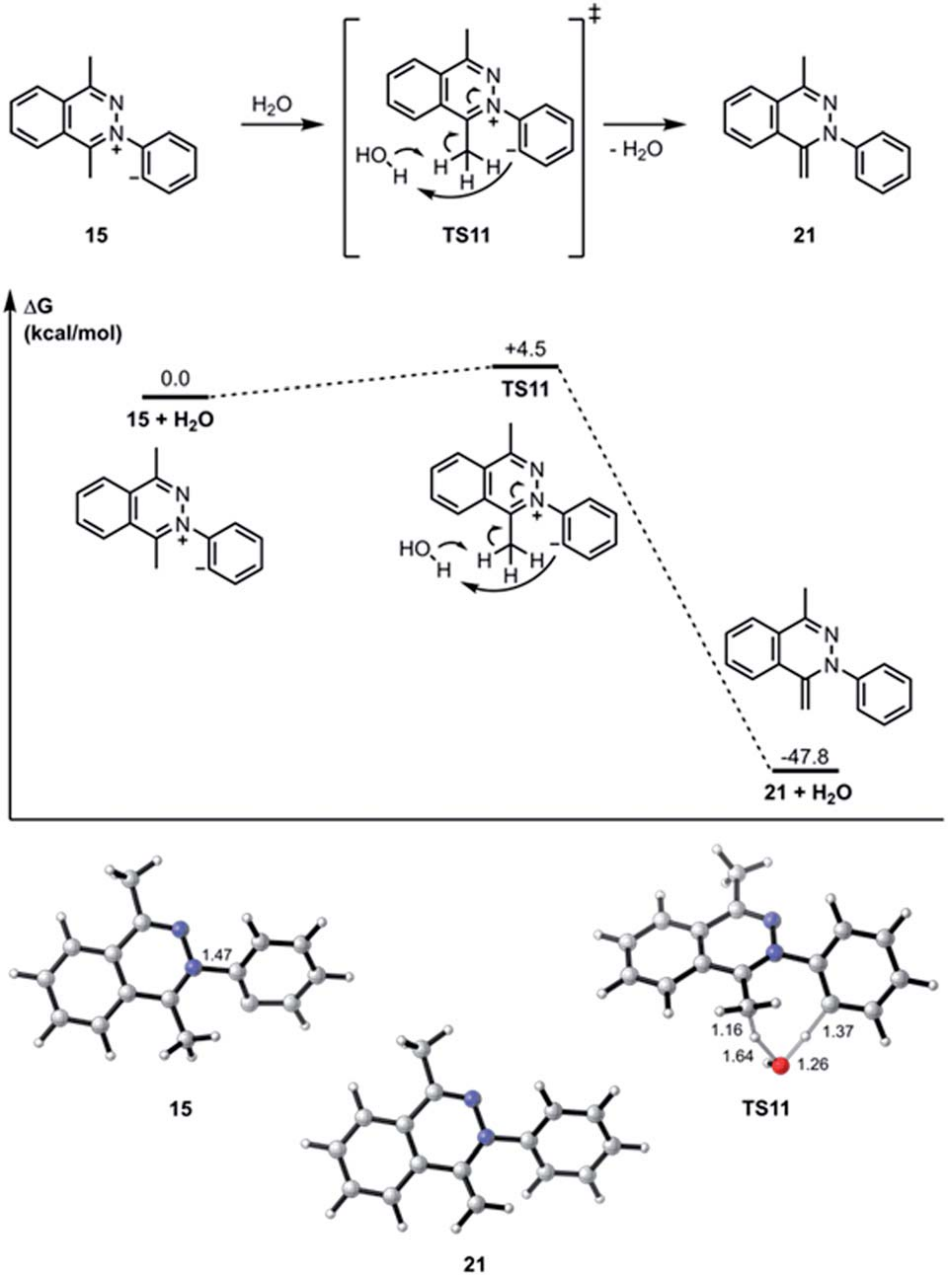
Optimized geometries of intermediates, products, and transition states for the water-assisted proton transfer pathway computed at the M06-2X/6-311+G(d,p)/SMD (CH_2_Cl_2_)//M06-2X/6-31G(d) level of theory. Gibbs free energies of activation are calculated with respect to isolated reactants and reported in kcal mol^–1^. Interatomic distances are shown in ångströms.

The transition state structure (**TS11**) for the concerted water-assisted proton transfer is shown in Fig. 5. The transition state for the direct intramolecular transfer of proton was not located. It is possible that due to the requirement that the σ C–H bond being broken in the proton abstraction must be in plane with the π system of the N=C bond, only a proton abstraction from the top or bottom face of the molecule would have been favorable. Due to the geometrical constraints of the molecular structure, however, such a top- or bottom-face proton abstraction by the anionic carbon is extremely difficult to achieve.

**Fig. 6:**
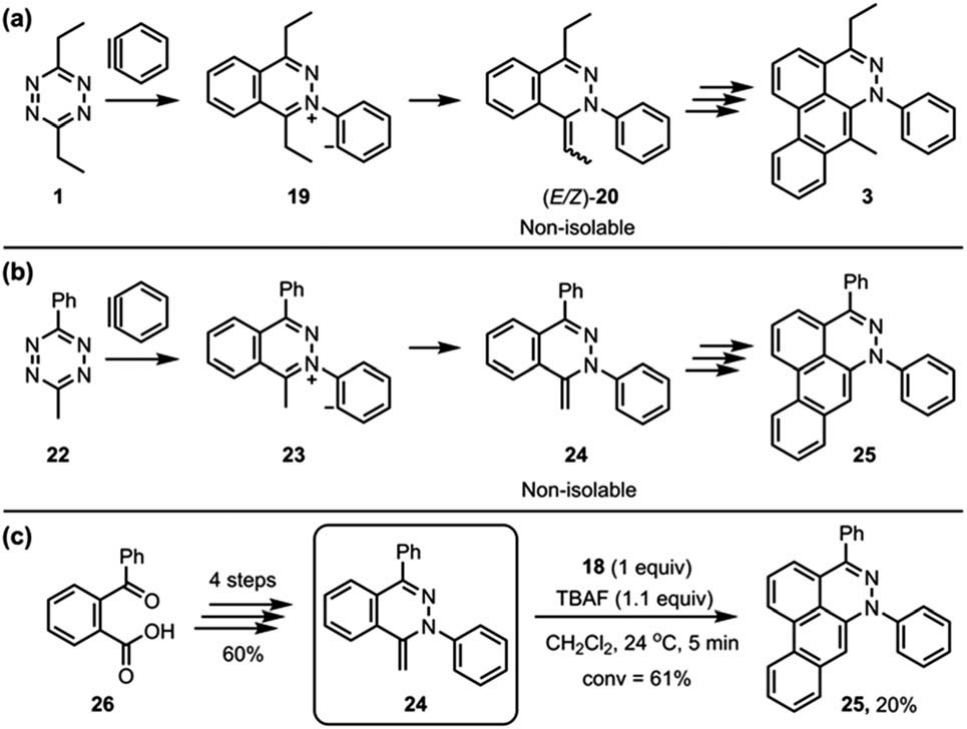
(a) The TAT reaction of 3,6-diethyltetrazine **1**. (b) Alternative probe **24** to the intermediates (*E/Z*)-**20 **of the TAT reaction. (c) The formation of **25** from the reaction of **24** and aryne.

To simplify the analysis of possible alkylidene intermediate, 3-methyl-6-phenyl-1,2,4,5-tetrazine (**24**) was employed (Fig. 6B); a phenyl group at the 6-position was expected to block the further side reaction and also stabilize the tetrazine ring, and a methyl group at the 3-position would afford a single methylidene intermediate **24** while 3,6-diethyltetrazine **1** could afford (*E*/*Z*)-**20** as an isomeric mixture (Fig. 6A). The aryne reaction of newly employed tetrazine **22** also did not stop at the intermediate **24**, instead proceeding to afford the dibenzo[*de*,*g*]cinnoline **25**. Even though **24** was not isolated, we were able to synthesize **24** through an alternate route (Fig. 6C).^12^ TBAF (1.1 equiv.) was subjected to a solution containing **24** (1.0 equiv.) and **18** (1.0 equiv.) in CH_2_Cl_2_, which afforded the desired product **25** in 20% yield. This result supports the notion that a methylidene intermediate, such as **24** or (*E*/*Z*)-**20**, might be the key intermediate in the third benzyne addition step of the TAT reaction.

**Fig. 7:**
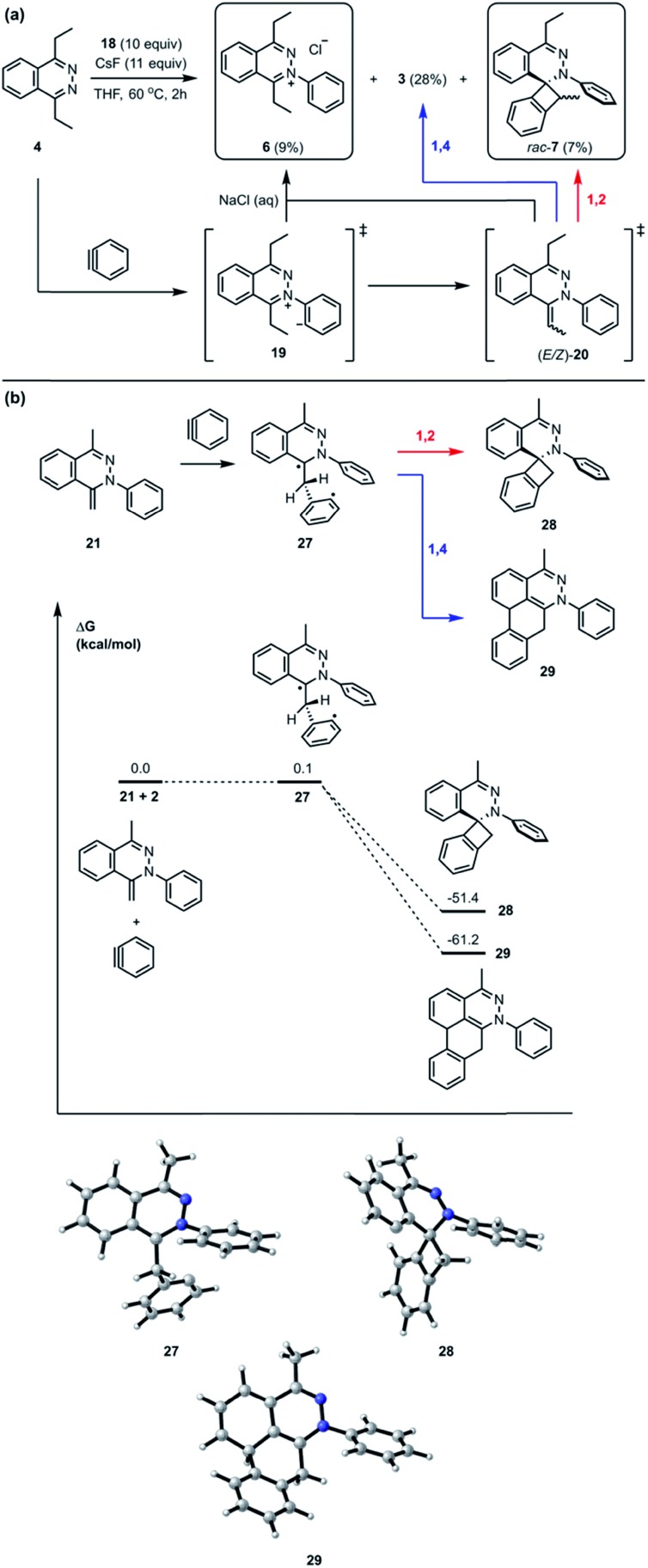
(a) The trapped intermediate salt **6** and competitive cycloaddition between [2 + 2] and [4 + 2]. (b) Optimized geometries of intermediates and product structures for [2 + 2] and [4 + 2] cycloaddition pathways from the intermediate **21** and aryne **2** computed at the M06-2X/6-311+G(d,p)/SMD (CH_2_Cl_2_)//M06-2X/6-31G(d) level of theory. Gibbs free energies are calculated with respect to isolated reactants, and reported in kcal mol^–1^.

Interestingly, the reaction between **4** and **18** with CsF in THF at 60 °C for 2 hours followed by treatment with saturated brine solution afforded the trapped intermediate **6** in 9% yield (Fig. 7A). There are two possible routes leading to the formation of **6**. One possibility is that the intermediate **19** is quenched by an external proton source such as a solvent molecule. The other possibility is that the intermediate **19** undergoes an intermolecular proton transfer to yield neutral intermediates (*E*/*Z*)-**20**, followed by tautomerization, to furnish hydrazonium salt **6**. The intermolecular pathway was ruled out in Fig. 4B. The [2 + 2] cycloaddition product *rac*-**7** was obtained as a mixture of inseparable diastereomers in 7% yield.^13^ The relatively smaller amounts of the [2 + 2] cycloaddition product compared to [4 + 2] product **3** indicate that the [4 + 2] mode is experimentally more favorable than the [2 + 2] mode. Calculations support the idea that the intermediate **21** can react with another equivalent of benzyne in either a formal [2 + 2] or [4 + 2] cycloaddition to give **28** and **29**, respectively (Fig. 7B). We performed calculations to elucidate whether the formal [2 + 2] and [4 + 2] cycloadditions occur *via* stepwise or concerted mechanisms, and whether they proceed through zwitterionic or diradical intermediates. A stepwise [2 + 2] pathway would be expected in light of the Woodward–Hoffmann rules, while a stepwise [4 + 2] is also likely for this system because aromaticity would need to be broken to form the σ C–C bond on one side but not the other, leading to very different energetic penalties. Indeed, transition states for concerted [2 + 2] or [4 + 2] cycloadditions were not found for this system. While all of the possible closed-shell zwitterionic intermediates for stepwise [2 + 2] and [4 + 2] pathways were found to be unstable, we located the common diradical intermediate **27** expected along stepwise pathways of both the formal [2 + 2] and [4 + 2] cycloadditions between **21** and **2**, with a free energy of +0.1 kcal mol^–1^. Gibbs free energies of the [2 + 2] adduct **28** and [4 + 2] adduct **29** indicate that these formal cycloadditions are strongly exergonic.

**Fig. 8:**
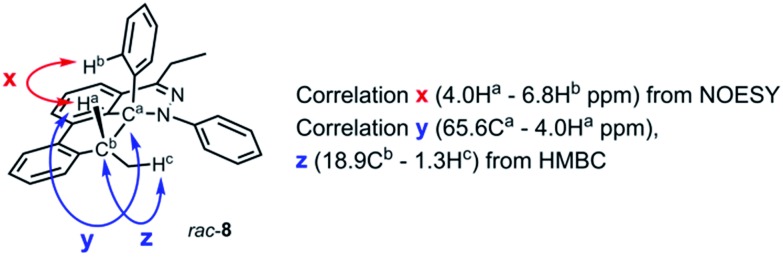
Representative NMR correlation assignments of *rac*-**8**.

During the analysis of the standard TAT reaction in Scheme 1, 8% of *rac*-**8** was isolated and characterized by HMBC NMR experiments, and its relative stereochemistry was determined by NOESY NMR experiments (Fig. 8). We propose two possible mechanisms for the synthesis of *rac*-**8** consisting of a concerted ene-reaction or stepwise ene-type reaction of *rac*-**30**.^14^ In both mechanisms, the two possible pathways support the existence of a currently non-isolable intermediate *rac*-**30**.

**Fig. 9:**
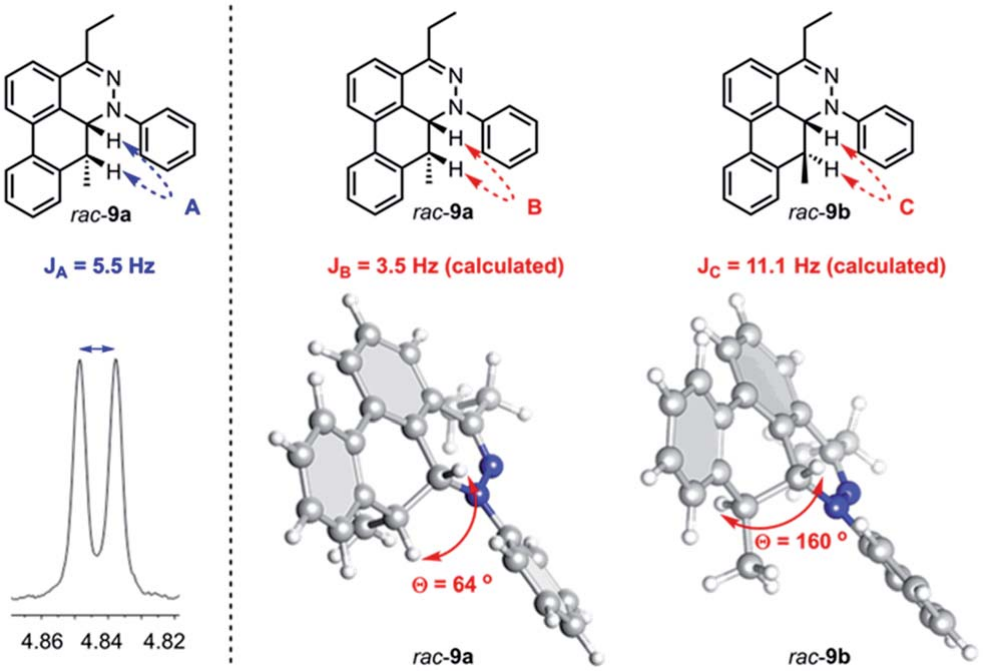
Determination of stereochemistry of *rac*-**9a**. *J* coupling constant of A from ^1^H NMR spectrum (left) and calculated *J* coupling constant and degree between two protons on *cis* intermediate *rac*-**9a** and *trans* intermediate *rac*-**9b** (right).

Further observation of the rearranged intermediate *rac*-**9a** indirectly supports that the intermediate *rac*-**30** is in the course of the TAT reaction. The structure of *rac*-**9a** was characterized by ^1^H NMR and HRMS spectroscopy. The *J* coupling constant of two vicinal protons (*J*_A_) was determined to be 5.5 Hz (Fig. 9). Based on the results from the B3LYP/IGLO-III//HF/6-31G(d) calculations, *cis* isomer *rac*-**9a** possessed 64° dihedral angles and a *J* coupling of 3.5 Hz between the two vicinal protons. Larger dihedral angles of 160° for the *trans* isomer (*rac*-**9b**) resulted in a *J*_C_ of 11.1 Hz, which is quite different from the observed value.

## Conclusions

We have provided a detailed mechanism of the reaction between a tetrazine and arynes. During the course of the reaction, three aryne addition steps occur consecutively with each step displaying a distinct reactivity mode with benzyne. By isolating and trapping the key intermediates, each of these distinct aryne reactivity modes as well as alternative side product pathways were elucidated. DFT calculations of transition states of two competitive reactions, further [4 + 2] cycloaddition and nucleophilic addition, support the assertion that [4 + 2] cycloaddition has higher activation energy barrier, and thus, the formation of anthracene is supposed to be unfavorable. The computational and experimental study for three plausible proton transfer pathways were performed. This analysis showed that the intermolecular water-assisted proton transfer is more favorable than the intramolecular proton transfer. The dibenzocinnoline **25** was observed during the reaction of an alternative intermediate **24** with an aryne *via* [4 + 2] cycloaddition. The intermediate **19** undergoes further formal [2 + 2] and [4 + 2] cycloaddition, with both the [2 + 2] cycloaddition product *rac*-**7** and the [4 + 2] product **3** being observed. In addition, the plausibility of non-isolable intermediates *rac*-**30** was confirmed by the isolation and characterization of the ene or the stepwise ene-type product *rac*-**8** and rearranged product *rac*-**9a**. The observed intermediates and byproducts are consistent with the overall proposed mechanism for the TAT reaction. We anticipate that the study of arynes–tetrazine-engaged reactivity modes will serve as guidance for future explorations of aryne chemistry, and especially in the context of intermolecular multiple consecutive aryne addition reactions.

## Conflicts of interest

There are no conflicts to declare.

## Supplementary Material

Supplementary information
