# Supplementary material for: The mechanism of the triple aryne–tetrazine reaction cascade: theory and experiment
Source: Chem Sci. 2018 Aug 23;9(39):7688–93. doi: 10.1039/c8sc01796d (PMC6238107; doi:10.1039/c8sc01796d)
Supplement: Supplementary file 1 [file SC-009-C8SC01796D-s001.pdf]

Electronic Supplementary Material (ESI) for Chemical Science.
This journal is © The Royal Society of Chemistry 2018

The Mechanism of the Triple Aryne-Tetrazine Reaction Cascade: Theory and Experiment

Sung-Eun Suh,[a] Shuming Chen,[b] K. N. Houk,[b] and David M. Chenoweth*[a]

[a.] Department of Chemistry, University of Pennsylvania, 231 South 34th Street, Philadelphia, Pennsylvania 19104, United States.

[b.] Department of Chemistry and Biochemistry, University of California, Los Angeles, California 90095-1569, United States.

Table of content

General Information S2

Experimental Procedures S2

References S9

NMR Spectra S10

Computational Data S24

General Information

All commercial reagents and solvents were used as received. Flash column chromatography was performed using Silicycle silica gel (55−65 Å pore diameter). Thin-layer chromatography was performed on Sorbent Technologies silica plates (250 μm thickness). Proton nuclear magnetic resonance spectroscopy (^{1}H NMR) and carbon nuclear magnetic resonance spectroscopy (^{13}C NMR) spectra were recorded on a Bruker DMX 500 ^{1}H NMR. High-resolution mass spectra were obtained by Dr. Rakesh Kohli at the University of Pennsylvania's Mass Spectrometry Service Center on a on a Waters LC-TOF mass spectrometer (model LCT-XE Premier) using electrospray ionization. X-ray diffraction data obtained and solved by Dr. Patrick Carroll at the University of Pennsylvania. High performance liquid chromatography analysis was performed using a Jasco HPLC instrument equipped with a Phenomenex column (Luna 5u C18(2) 100A; 250 × 4.60 mm, 5 μm).

Computational Methods

For all computed structures other than *rac*-**9a** and *rac*-**9b**:

All computations were performed with the Gaussian 09[1] suite of programs. Ground state and transition state geomet ries were optimized in the gas phase using the M06-2X[2] functional using the 6-31G(d) basis set. Frequency calcula tions were performed at the same level of theory to determine whether the optimized structures are minima (no im aginary frequencies) or saddle points (one imaginary frequency) on the potential energy surface, and to obtain ther mal corrections to the Gibbs free energies. Single point electronic energies were computed with the M06-2X functi onal using the 6-311G++(d,p) basis set. Solvation effects were included using the SMD[3] solvation model (solvent=d ichloromethane). Gibbs free energies reported include zero-point energies and thermal corrections calculated at 298.1 5 K and 1 atm. Molecular structures were visualized using CYLview.[4]

For *rac*-**9a** and *rac*-**9b**:

Spin-spin coupling constant calculations were performed using Gaussian 09 at the B3LYP/IGLO-III//HF/6-31G(d) level of theory. Post processing was performed using UCSF Chimera.[5]

Experimental Procedures

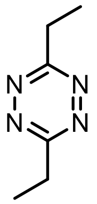

1

3,6-diethyl-1,2,4,5-tetrazine (1): Synthetic method and characterization data of **1** have been reported in our previous publication.[6]

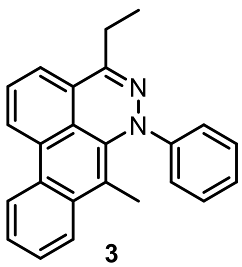

3

4-ethyl-7-methyl-6-phenyl-6H-dibenzo[de,g]cinnoline (3): Synthetic method and characterization data of **3** have been reported in our previous publication.[6]

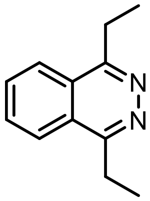

4

1,4-diethylphthalazine (4): Synthetic method and characterization data of **4** have been reported in our previous publication.[7]

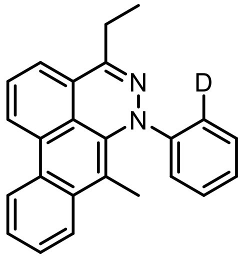

[D]-**3**

4-ethyl-7-methyl-6-(phenyl-2-*d*)-6*H*-dibenzo[*de,g*]cinnoline ([D]-3): A 10 mL round-bottom flask was charged with 43 µmol of phthalazine **4** (8.0 mg), 430 µmol of 2-(trimethylsilyl)phenyl trifluoromethanesulfonate **18** (128 mg), 470 µmol of anhydrous CsF (72 mg) in 40 µL of CD_3CN and 40 µL of D_2O and was heated under reflux. After 2 hours, the solution was cooled down, concentrated in vacuo and purified by flash column chromatography (ethyl acetate/hexane 1:30) to afford [D]-**3** (1.6 mg).

Isolated Yield: 11 %.

Physical Property: Yellow solid, m.p. = 59-60 ˚C.

TLC: $\mathbf{R_f}$ = 0.81 (silica gel, ethyl acetate/hexanes 1:4).

^{1}H NMR (500 MHz, CD_2Cl_2) δ 8.53-8.49 (m, 2H), 7.86 (dd, 1H, J = 8.2, 1.3 Hz), 7.63 (dd, 1H, J = 8.2, 7.4 Hz), 7.58 (ddd, 1H, J = 8.2, 6.9 1.3 Hz), 7.48 (ddd, 1H, J = 8.2, 6.9, 1.3 Hz), 7.43 (dd, 1H, J = 7.4, 1.0 Hz), 7.39-7.33 (m, 3H), 7.12-7.07 (m, 1H), 2.82 (q, 2H, J = 7.3 Hz), 2.00 (s, 3H), 1.36 (t, 3H, J = 7.3 Hz)

HRMS (ESI) calculated for $C_{24}H_{19}DN_2^+$ $[M]^+$ 337.1684, found 337.1689.

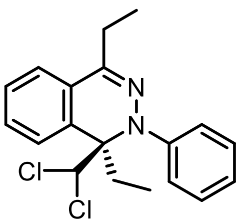

rac-**5a**

(*R*)- and (*S*)-1-(dichloromethyl)-1,4-diethyl-2-phenyl-1,2-dihydrophthalazine (*rac*-5a): Synthetic method and characterization data of *rac*-**5a** have been reported in our previous publication.[7]

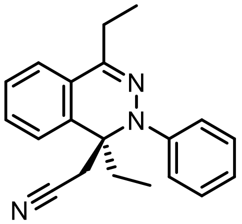

rac-**5b**

(*R*)- and (*S*)-2-(1,4-diethyl-2-phenyl-1,2-dihydrophthalazin-1-yl)acetonitrile (*rac*-5b): Synthetic method and characterization data of *rac*-**5b** have been reported in our previous publication.[7]

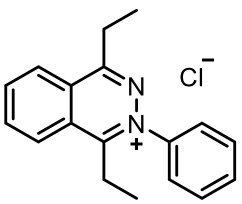

6

1,4-diethyl-2-phenylphthalazin-2-ium chloride (6): A 4 mL vial was charged with 48 μmol of 1,4-diethylphthalazine **4** (9.0 mg), 480 μmol of 2-(trimethylsilyl)phenyl trifluoromethanesulfonate **18** (144 mg), 530 μmol of anhydrous CsF (81 mg) in 0.05 mL of THF was heated to 60 ºC. After 2 hours, the solution was cooled down, concentrated in vacuo and purified by reversed phase column chromatography using water containing 0.1% trifluoroacetic acid. Trifluoroacetate ion was replaced with chloride ion by the addition of a drop of brine to the resulting solid followed by co-evaporation under high-vacuum condition. The solid was filtered with HPLC grade dichloromethane and the filtrate was dried in vacuo for 12 hours to afford **6** (1.3 mg).

Isolated Yield: 9 %.

Physical Property: Colorless semi-solid.

^{1}H NMR (500 MHz, CD_2Cl_2)

δ 8.74 (d, 1H, J = 8.3 Hz), 8.55 (d, 1H, J = 8.3 Hz), 8.48 (ddd, 1H, J = 8.3, 7.2, 1.1 Hz), 8.40 (ddd, 1H, J = 8.3, 7.2, 1.1 Hz), 7.80-7.72 (m, 3H), 7.68-7.63 (m, 2H), 3.56-3.48 (m, 4H), 1.50 (t, 3H, J = 7.4 Hz), 1.45 (t, 3H, J = 7.7 Hz).

^{13}C NMR (125 MHz, CD_2Cl_2)

δ 165.7, 164.6, 142.6, 139.1, 136.0, 131.5, 130.3, 129.3, 127.5, 127.2, 126.4, 125.1, 26.3, 25.1, 14.1, 11.5.

IR (neat): 2917, 2849, 1265, 1154, 1031, 638 cm^{-1}.

HRMS (ESI) calculated for $C_{18}H_{19}N_2^+$ [M] 263.1543, found 263.1544.

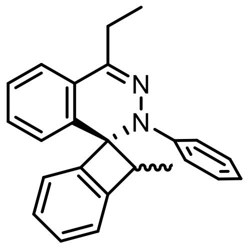

rac-**7**

(7R,8R)-, (7S,8S)-, (7R,8S)-, and (7S,8R)-4'-ethyl-8-methyl-2'-phenyl-2'H-spiro[bicyclo[4.2.0]octane-7,1'-phthalazine]-1(6),2,4-triene (*rac*-7): A 4 mL vial was charged with 48 μmol of 1,4-diethylphthalazine **4** (9.0 mg), 480 μmol of 2-(trimethylsilyl)phenyl trifluoromethanesulfonate **18** (144 mg), 530 μmol of anhydrous CsF (81 mg) in 50 μL of THF was heated to 60 °C. After 2 hours, the solution was cooled down, concentrated in vacuo and purified by reversed phase column chromatography using water containing 0.1% trifluoroacetic acid to afford 1.1 mg of *rac*-**7**.

Isolated Yield: 7 % (overall).

Physical Property: White solid.

TLC: R$_f$ = 0.30 (silica gel, ethyl acetate/hexanes 1:4).

^{1}H NMR (500 MHz, $CDCl_3$)

δ 7.71-7.67 (m, Major 1H), 7.44-6.96 (m, 10H Major, 11H Minor), 6.84 (td, Major 1H, J = 7.4, 0.9 Hz), 6.82 (td, Minor 1H, J = 7.4, 0.9 Hz), 6.66 (d, Minor 1H, J = 7.8 Hz), 6.54 (d, Major 1H, J = 7.8 Hz), 3.87 (q, Major 1H, J = 7.0 Hz), 3.53 (q, Minor 1H, J = 7.3 Hz), 2.82-2.62 (m, 2H Major, 2H Minor), 1.30 (t, 3H Minor, J = 7.4 Hz), 1.24 (t, 3H Major, J = 7.4 Hz), 1.05 (d, 3H Minor, J = 7.3 Hz), 0.94 (d, 3H Major, J = 7.0 Hz).

IR (neat): 2963, 2921, 2851, 1723, 1596, 1495, 1477, 1459, 1093, 1070, 1044, 800, 743, 699 cm^{-1}.

HRMS (ESI) calculated for $C_{24}H_{23}N_2^+$ $[M+H]^+$ 339.1856, found 339.1864.

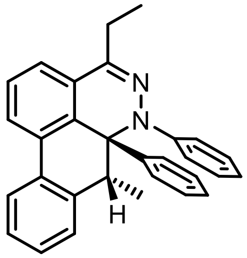

rac-**8**

(6a*S*,7*R*)- and (6a*R*,7*S*)-4-ethyl-7-methyl-6,6a-diphenyl-6a,7-dihydro-6*H*-dibenzo[*de,g*]cinnoline (*rac*-8): A 4 mL vial was charged with 48 μmol of 1,4-diethylphthalazine **4** (9.0 mg), 0.483 mmol of 2-(trimethylsilyl)phenyl trifluoromethanesulfonate **18** (144 mg) in 50 μL of dichloromethane at 24 °C, 1.0 M TBAF in THF (530 μmol, 530 μL) was slowly added to the solution over the course of 5 min. After the addition, the solution was concentrated in vacuo, and filtered on the silica gel, and purified by flash column chromatography (ethyl acetate/hexanes 1:20) to afford *rac*-**8** (1.6 mg).

Isolated Yield: 8 %.

Physical Property: Colorless oil.

TLC: R$_f$ = 0.62 (silica gel, ethyl acetate/hexanes 1:4).

^{1}H NMR (500 MHz, $CDCl_3$)

δ 7.80-7.75 (m, 1H), 7.59-7.56 (m, 1H), 7.52-7.49 (m, 2H), 7.26-7.22 (m, 1H), 7.22-7.15 (m, 4H), 7.14-7.08 (m, 1H), 7.02-6.92 (m, 5H), 6.83-6.79 (m, 2H), 4.01 (q, 1H, J = 6.7 Hz), 2.90-2.68 (m, 2H), 1.48 (t, 3H, J = 6.7 Hz), 1.30 (t, 3H, J = 7.5 Hz).

^{13}C NMR (125 MHz, $CDCl_3$)

δ 147.2, 142.8, 140.9, 140.6, 132.1, 131.8, 130.6, 128.5, 128.2, 128.1, 127.8, 127.6, 127.1, 127.0, 126.9, 126.1, 125.8, 125.3, 124.7, 123.7, 122.4, 65.6, 40.7, 25.7, 18.9, 11.8.

IR (neat): 2963, 2919, 2849, 1259, 1085, 1018, 795, 704 cm^{-1}.

HRMS (ESI) calculated for $C_{30}H_{27}N_2^+$ $[M+H]^+$ 415.2169, found 415.2174.

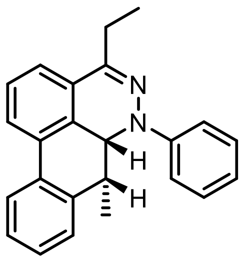

rac-**9a**

(6a*R*,7*R*)- and (6a*S*,7*S*)-4-ethyl-7-methyl-6-phenyl-6a,7-dihydro-6*H*-dibenzo[*de,g*]cinnoline (*rac*-9a): A 4 mL vial was charged with 48 μmol of 1,4-diethylphthalazine **4** (9.0 mg), 480 mmol of 2-(trimethylsilyl)phenyl trifluoromethanesulfonate **18** (144 mg) in 50 μL of dichloromethane at 24 ºC, 1.0 M TBAF in THF (530 μmol, 530 μL) was slowly added to the solution over the course of 5 min. After the addition, the solution was concentrated in vacuo, and filtered on the silica gel, and purified by flash column chromatography (ethyl acetate/hexanes 1:20) to afford *rac*-**9a** (0.1 mg). The extremely low stability and yield precluded the ^{13}C NMR characterization.

Isolated Yield: Less than 1 %.

Physical Property: White semi-solid.

TLC: R$_f$ = 0.56 (silica gel, ethyl acetate/hexanes 1:4).

^{1}H NMR (500 MHz, $CDCl_3$)

δ 7.70 (d, 1H, J = 7.9 Hz), 7.50-7.23 (m, 11H), 4.84 (d, 1H, J = 5.5 Hz), 3.54-3.46 (m, 1H), 2.52-2.41 (m, 2H), 1.35 (d, 3H, J = 7.5 Hz), 0.95 (t, 3H, J = 7.4 Hz).

IR (neat): 2968, 2926, 2854, 1259, 1096, 1026, 800 cm^{-1}.

HRMS (ESI) calculated for $C_{24}H_{23}N_2^+$ $[M+H]^+$ 339.1856, found 339.1865.

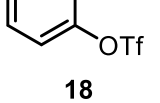

18

2-(trimethylsilyl)phenyl trifluoromethanesulfonate (18): 18 was prepared according to the literature procedure[8] and characterization data of **18** has been reported in our previous publication.[7]

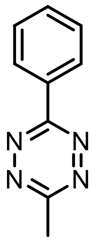

22

3-methyl-6-phenyl-1,2,4,5-tetrazine (22): Synthetic method and characterization data of **22** have been reported in our previous publication.[6]

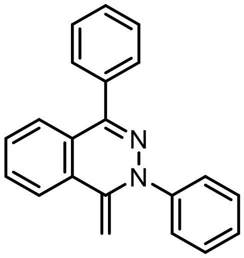

24

1-methylene-2,4-diphenyl-1,2-dihydrophthalazine (24): 3.0 M methylmagnesium bromide solution (0.3 mmol, 0.3 mL) in diethyl ether was slowly added to 0.1 mmol of 2,4-diphenyl-1(2*H*)-phthalazinone[9] (30 mg) in 0.3 mL of benzene at 24 °C. After 10 hours, 1M HCl solution was added.[10] Then, K_2CO_3 was added until the pH of the solution became 7. The organic layer was extracted with dichloromethane and water, was washed with brine solution, and was purified by flash column chromatography (ethyl acetate/hexanes 1:1 to 9:1) to afford **24** (23 mg).

Isolated Yield: 77 %

Physical Property: Green oil.

TLC: R$_f$ = 0.30 (silica gel, ethyl acetate/hexanes 1:4).

^{1}H NMR (500 MHz, $CDCl_3$)

δ 7.77 (d, 1H, J = 7.9 Hz), 7.64-7.58 (m, 4H), 7.54-7.44 (m, 6H), 7.427.37 (m, 2H), 7.31 (t, 1H, J = 7.4 Hz), 4.52 (s, 1H), 4.17 (s, 1H).

^{13}C NMR (125 MHz, $CDCl_3$)

δ 145.3, 144.1, 142.4, 136.1, 131.9, 131.0, 129.3, 129.11, 129.09, 128.4, 128.3, 126.2, 125.8, 125.5, 124.7, 123.9, 81.6.

IR (neat): 2965, 2921, 2848, 1661, 1594, 1492, 1443, 1259, 1096, 1021, 797, 696 cm^{-1}.

HRMS (ESI) calculated for $C_{21}H_{17}N_2^+$ $[M+H]^+$ 297.1386, found 297.1404.

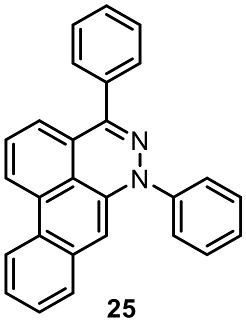

25

4,6-diphenyl-6*H*-dibenzo[*de*,g]cinnoline (25): A vial was charged with 24 µmol of **16** (7.0 mg), 24 µmol of 2-(trimethylsilyl)phenyl trifluoromethanesulfonate **18** (7.2 mg) in 30 µL of dichloromethane at 24˚C, and 1.0 M TBAF in THF (26 µmol, 26 µL) was slowly added to the solution over the course of 1 min. After the addition, the solution was concentrated in vacuo and purified by flash column chromatography (ethyl acetate/hexane 1:30) to afford **25** (1.8 mg) in 20% yield. Characterization data of **25** have been reported in our previous publication.[6]

Deuterium Labeling Experiment in Figure 4B and 3C.

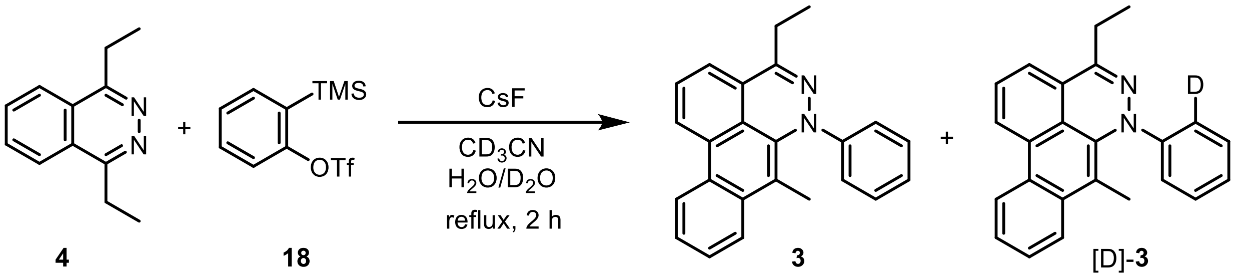

Each 10 mL round-bottom flask was charged with 43 µmol of phthalazine **4** (8.0 mg), 430 µmol of 2-(trimethylsilyl)phenyl trifluoromethanesulfonate **18** (128 mg), 470 µmol of anhydrous CsF (72 mg) in 40 µL of CD_3CN and 40 µL of D_2O (or 40 µL of CD_3CN and 40 µL of H_2O) and was heated under reflux. After 2 hours, the solution was cooled down, concentrated in vacuo and purified by flash column chromatography (ethyl acetate/hexane 1:30) to afford **3** (or [D]-**3**).

References

1) M. J. Frisch, G. W. Trucks, H. B. Schlegel, G. E. Scuseria, M. A. Robb, J. R. Cheeseman, G. Scalmani, V. Barone, B. Mennucci, G. A. Petersson, H. Nakatsuji, M. Caricato, X. Li, H. P. Hratchian, A. F. Izmaylov, J. Bloino, G. Zheng, J. L. Sonnenberg, M. Hada, M. Ehara, K. Toyota, R. Fukuda, J. Hasegawa, M. Ishida, T. Nakajima, Y. Honda, O. Kitao, H. Nakai, T. Vreven, J. A. Montgomery, Jr., J. E. Peralta, F. Ogliaro, M. Bearpark, J. J. Heyd, E. Brothers, K. N. Kudin, V. N. Staroverov, R. Kobayashi, J. Normand, K. Raghavachari, A. Rendell, J. C. Burant, S. S. Iyengar, J. Tomasi, M. Cossi, N. Rega, J. M. Millam, M. Klene, J. E. Knox, J. B. Cross, V. Bakken, C. Adamo, J. Jaramillo, R. Gomperts, R. E. Stratmann, O. Yazyev, A. J. Austin, R. Cammi, C. Pomelli, J. W. Ochterski, R. L. Martin, K. Morokuma, V. G. Zakrzewski, G. A. Voth, P. Salvador, J. J. Dannenberg, S. Dapprich, A. D. Daniels, Ö. Farkas, J. B. Foresman, J. V. Ortiz, J. Cioslowski, and D. J. Fox, Gaussian 09, Revision B.01.; Gaussian, Inc.: Wallingford, CT, 2009.

2) Y. Zhao, D. G. Truhlar, *Theor. Chem. Acc.,* 2008, **120**, 215–241.

3) A. V. Marenich, C. J. Cramer, D. G. Truhlar, *J. Phys. Chem. B,* 2009, **113**, 6378.

4) C. Y. Legault, CYLview, 1.0b, Université de Sherbrooke, 2009 (http://www.cylview.org).

5) E. F. Pettersen, T. D. Goddard, C. C. Huang, G. S. Couch, D. M. Greenblatt, E. C. Meng, T. E. Ferrin, *J. Comput. Chem.,* 2004, **25**, 1605–1612.

6) S.-E. Suh, S. A. Barros, D. M. Chenoweth, *Chem. Sci.,* 2015, **6**, 5128–5132.

7) S.-E. Suh, D. M. Chenoweth, *Org. Lett.*, 2016, **18**, 4080–4083.

8) B. S. Shaibu, R. K. Kawade, R.-S. Liu, *Org. Biomol. Chem.,* 2012, **10**, 6834–6839.

9) M. M. Hemdan, S. M. Taha, A. M. Gabr, M. Y. Elkady, *J. Chem. Res.,* 2010, **34**, 102–105.

10) I. G. Ovchinnikova, I. V. Samartseva, E. A. Pavlova, *Zh. Org. Khim.*, 1984, **20**, 2248–2249.

NMR Spectra

^{1}H NMR spectrum of *rac*-[D_1]-**3b** in CD_2Cl_2 (500 MHz).

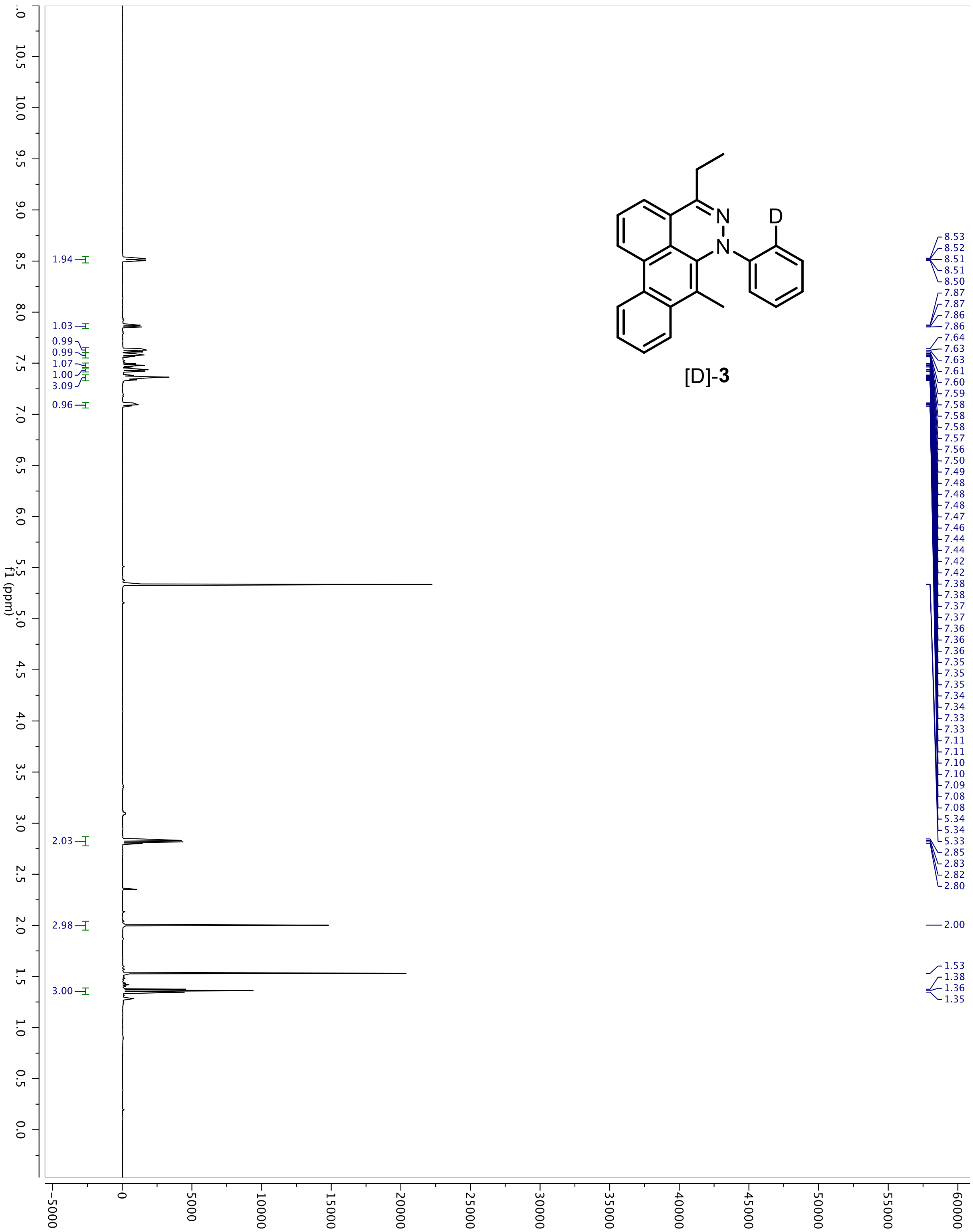

^{1}H NMR spectrum of **6** in CD_2Cl_2 (500 MHz).

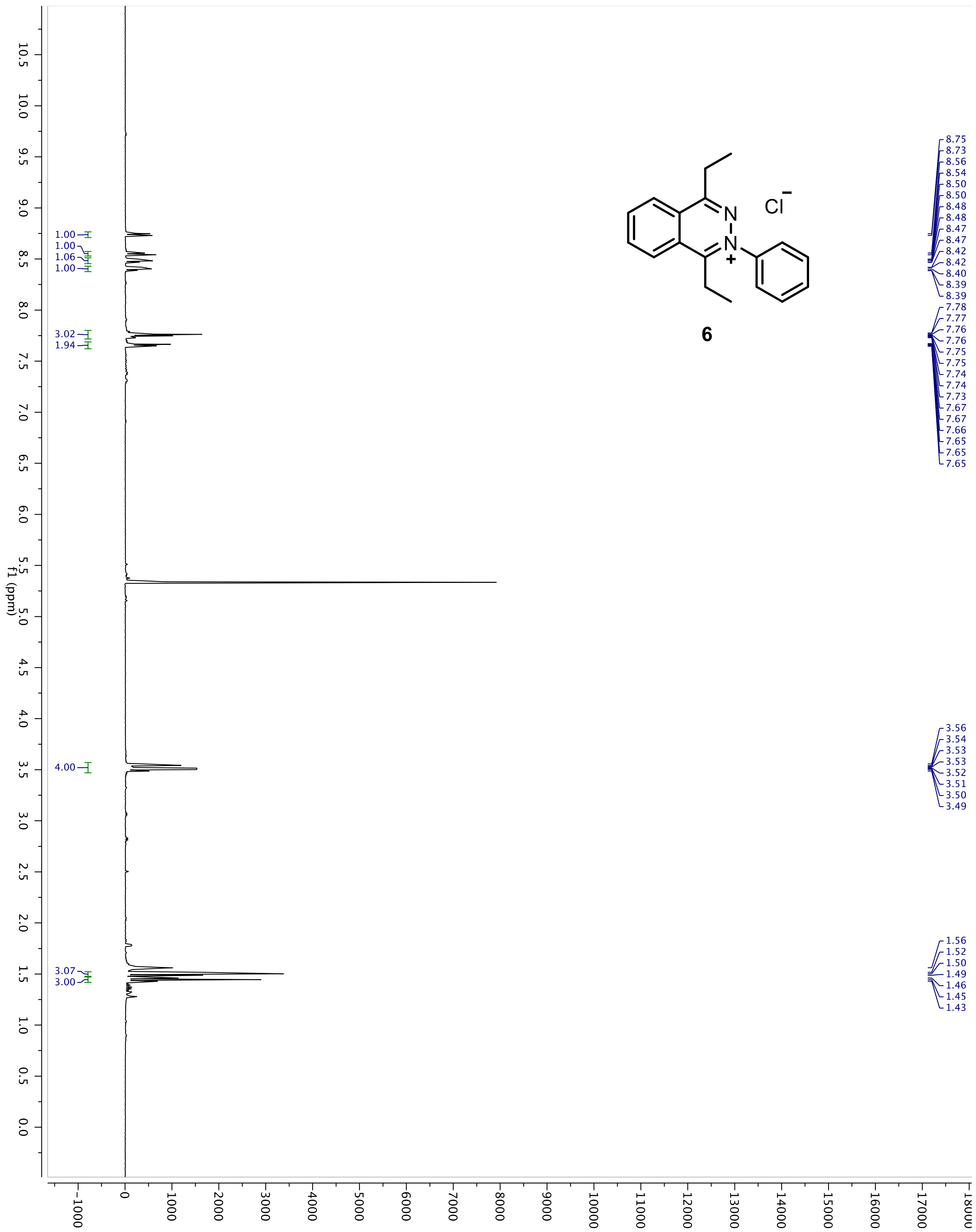

^{13}C NMR spectrum of **6** in CD_2Cl_2 (125 MHz).

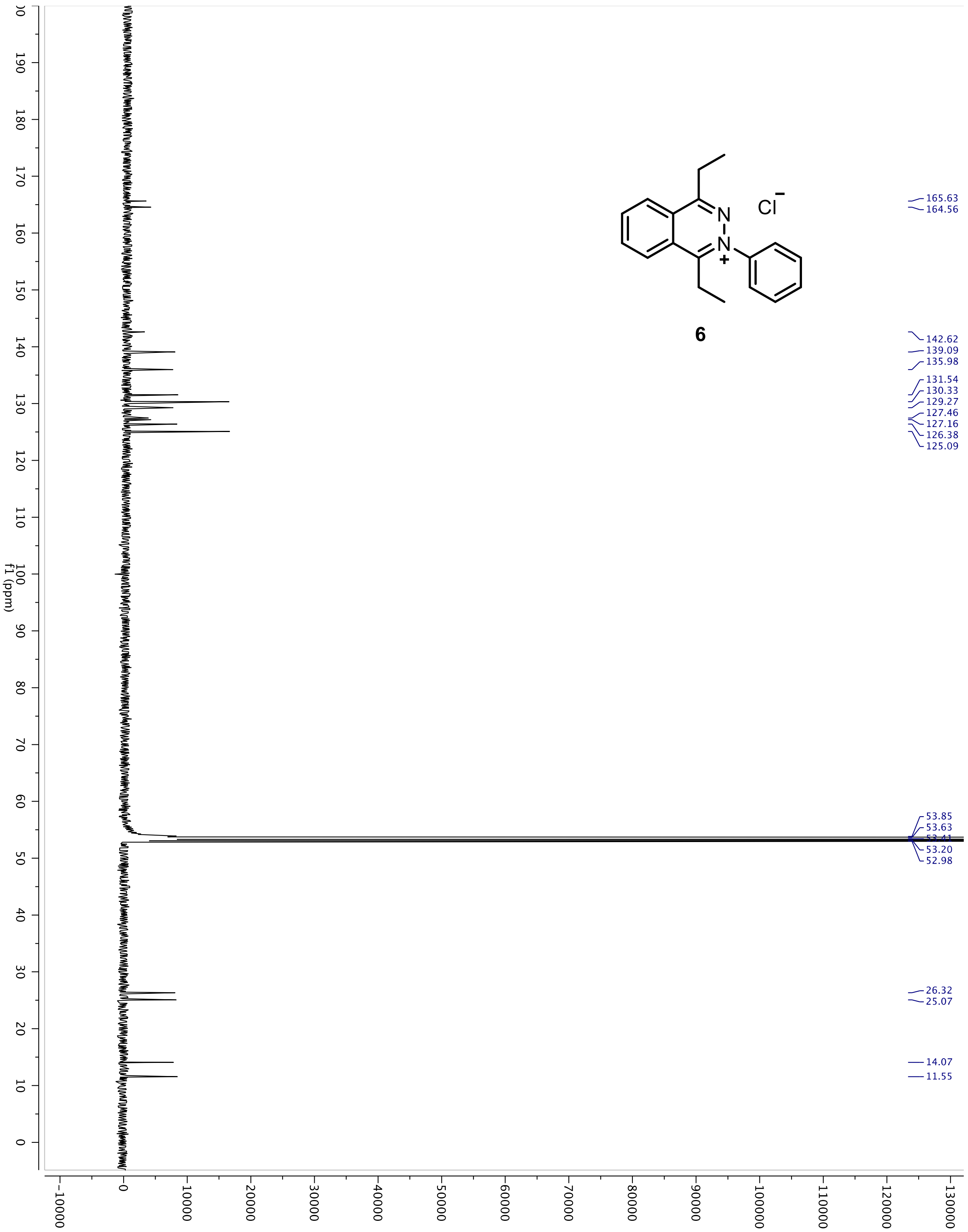

^{1}H NMR spectrum of mixture *rac*-**7** in CD_2Cl_2 (500 MHz).

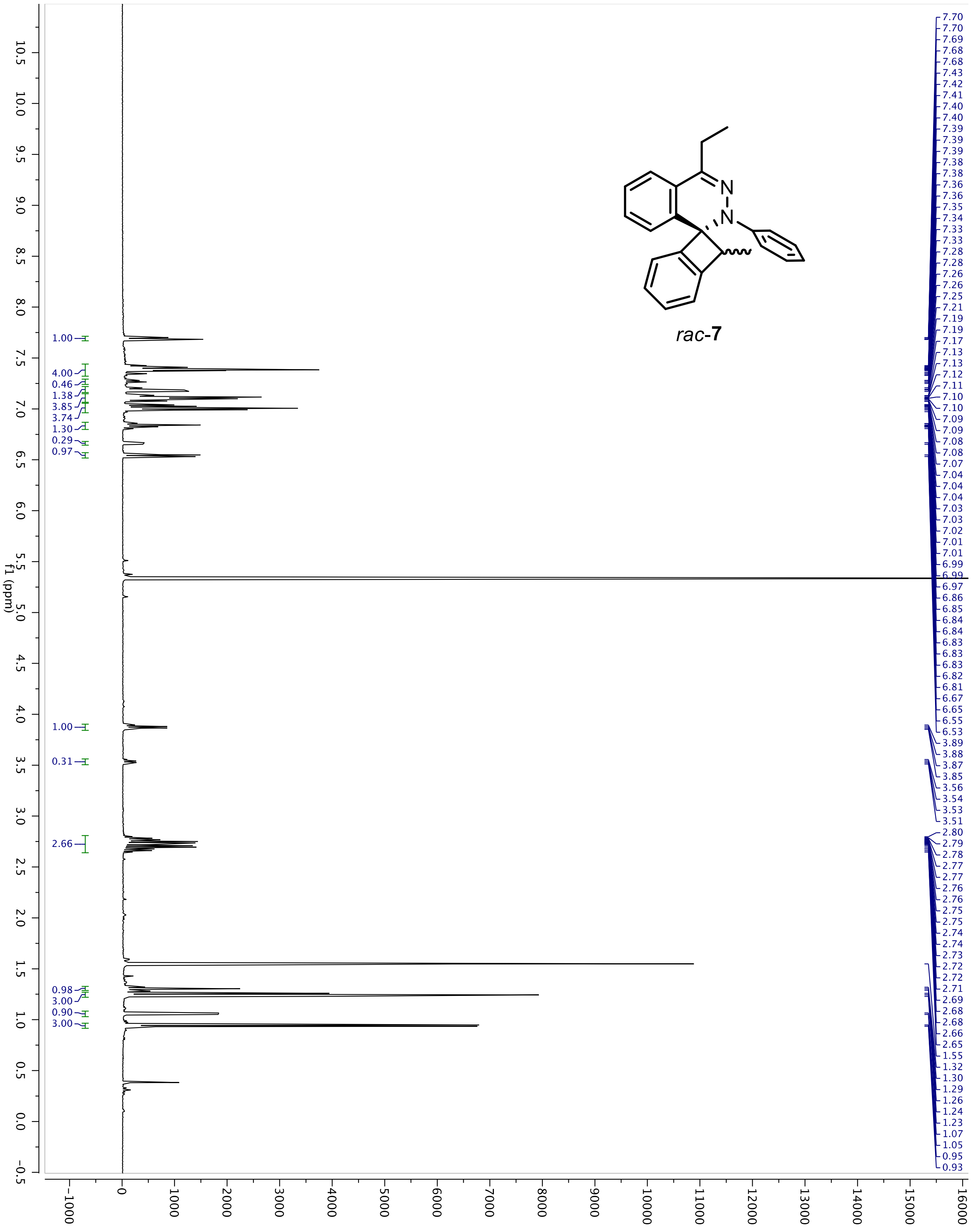

1st expanded ^{1}H NMR spectrum of mixture *rac*-**7** in CD_2Cl_2 (500 MHz).

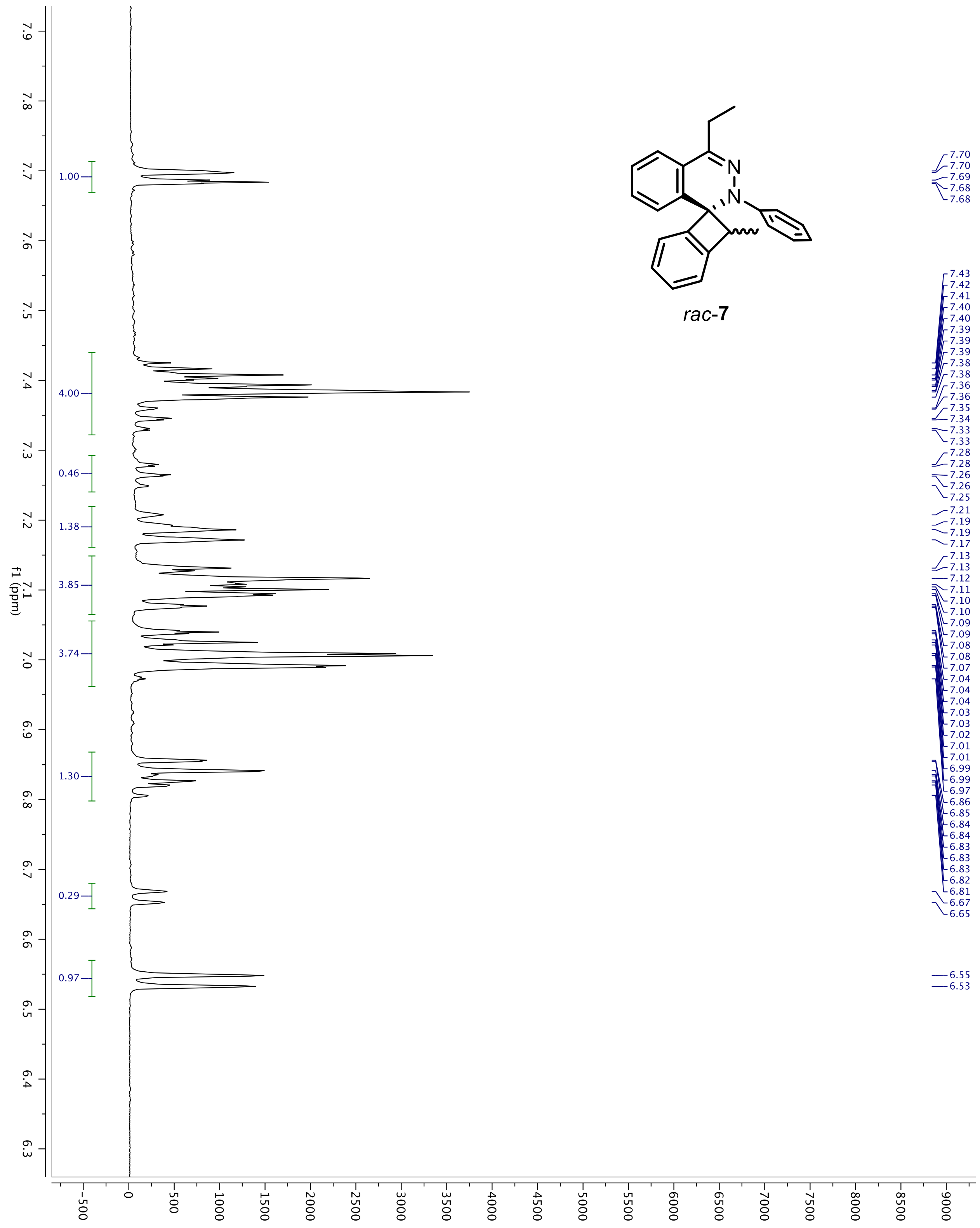

2nd expanded ^{1}H NMR spectrum of mixture *rac*-**7** in CD_2Cl_2 (500 MHz).

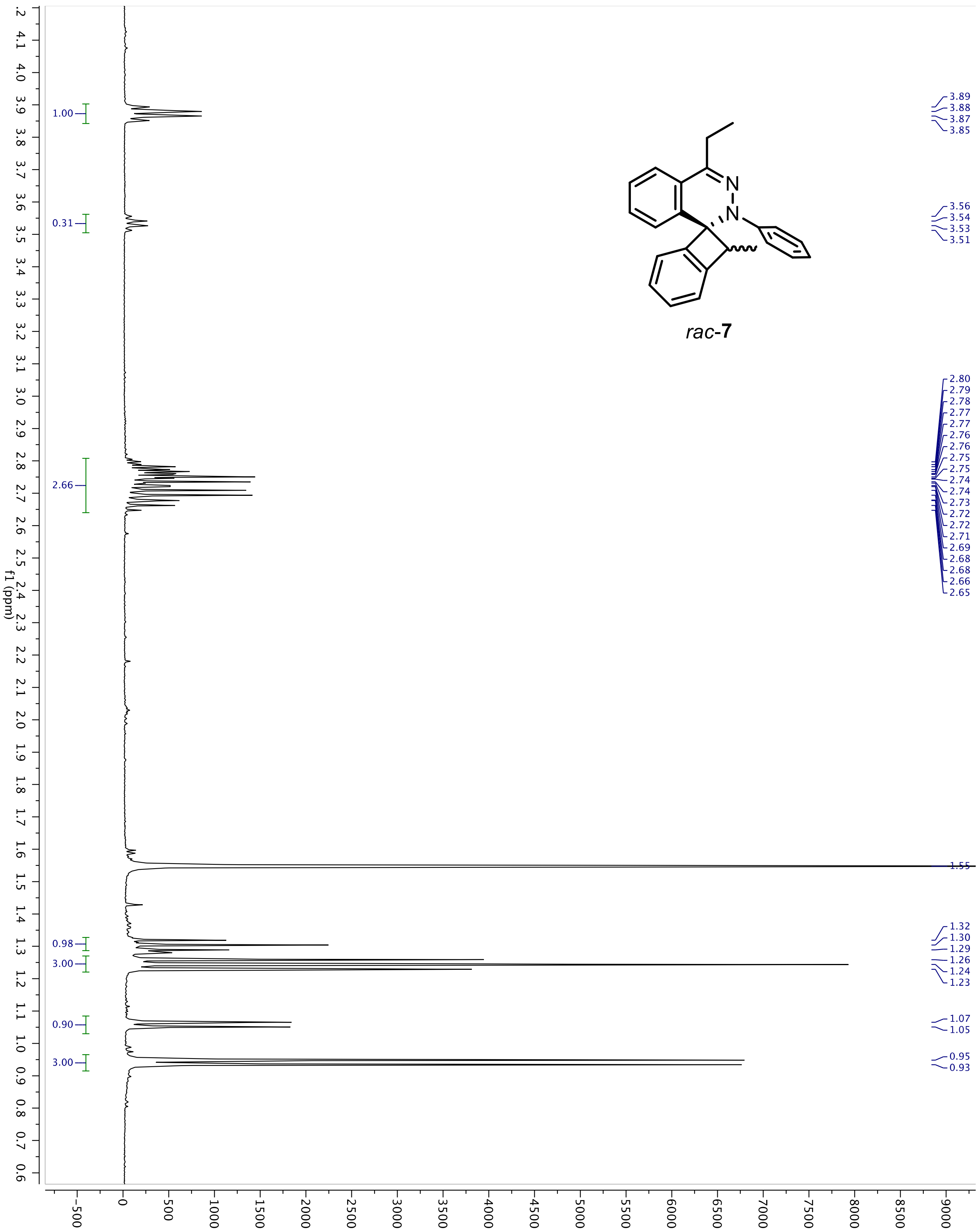

^{1}H NMR spectrum of *rac*-**8** in CD_2Cl_2 (500 MHz).

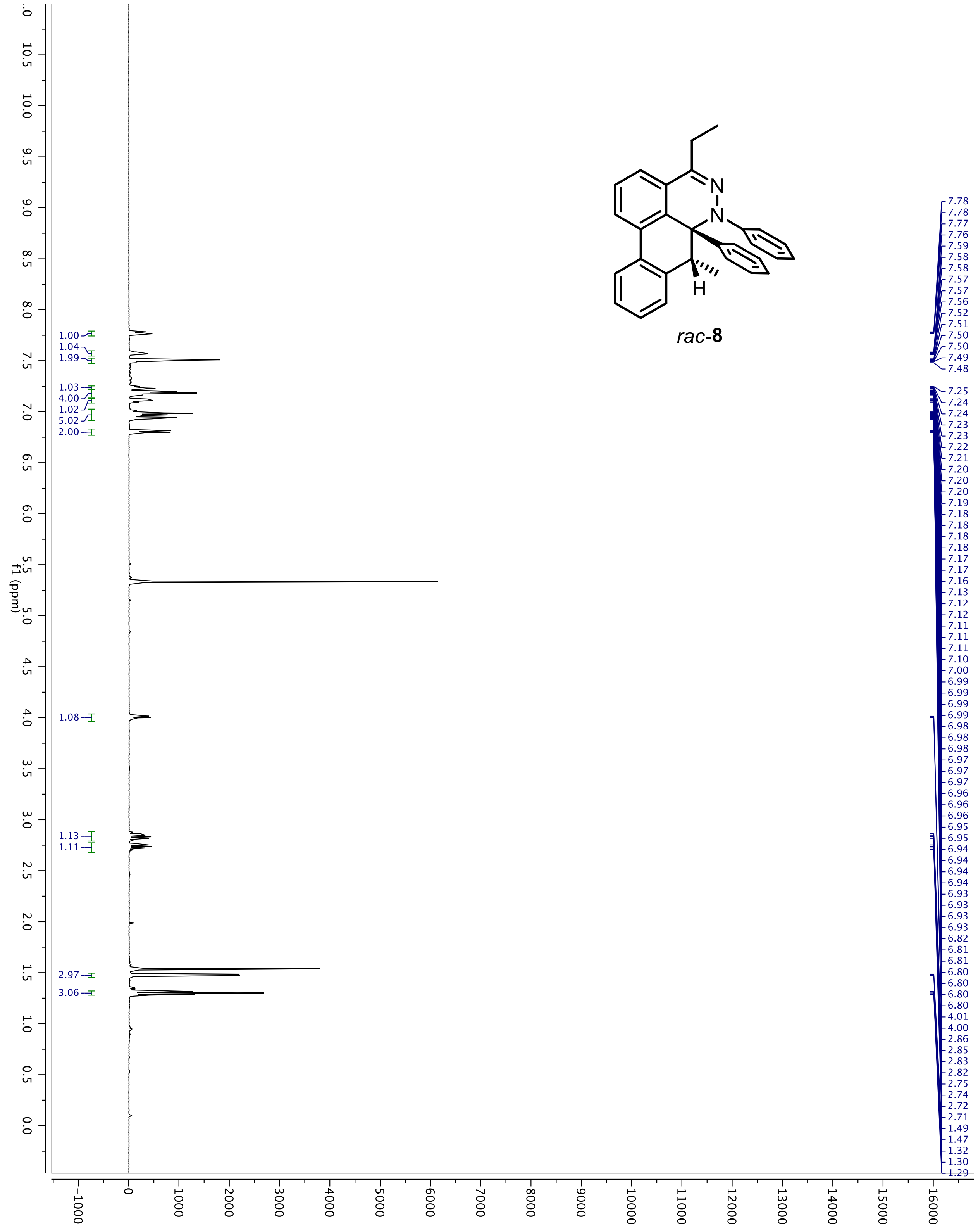

^{13}C NMR spectrum of *rac*-**8** in CD_2Cl_2 (125 MHz).

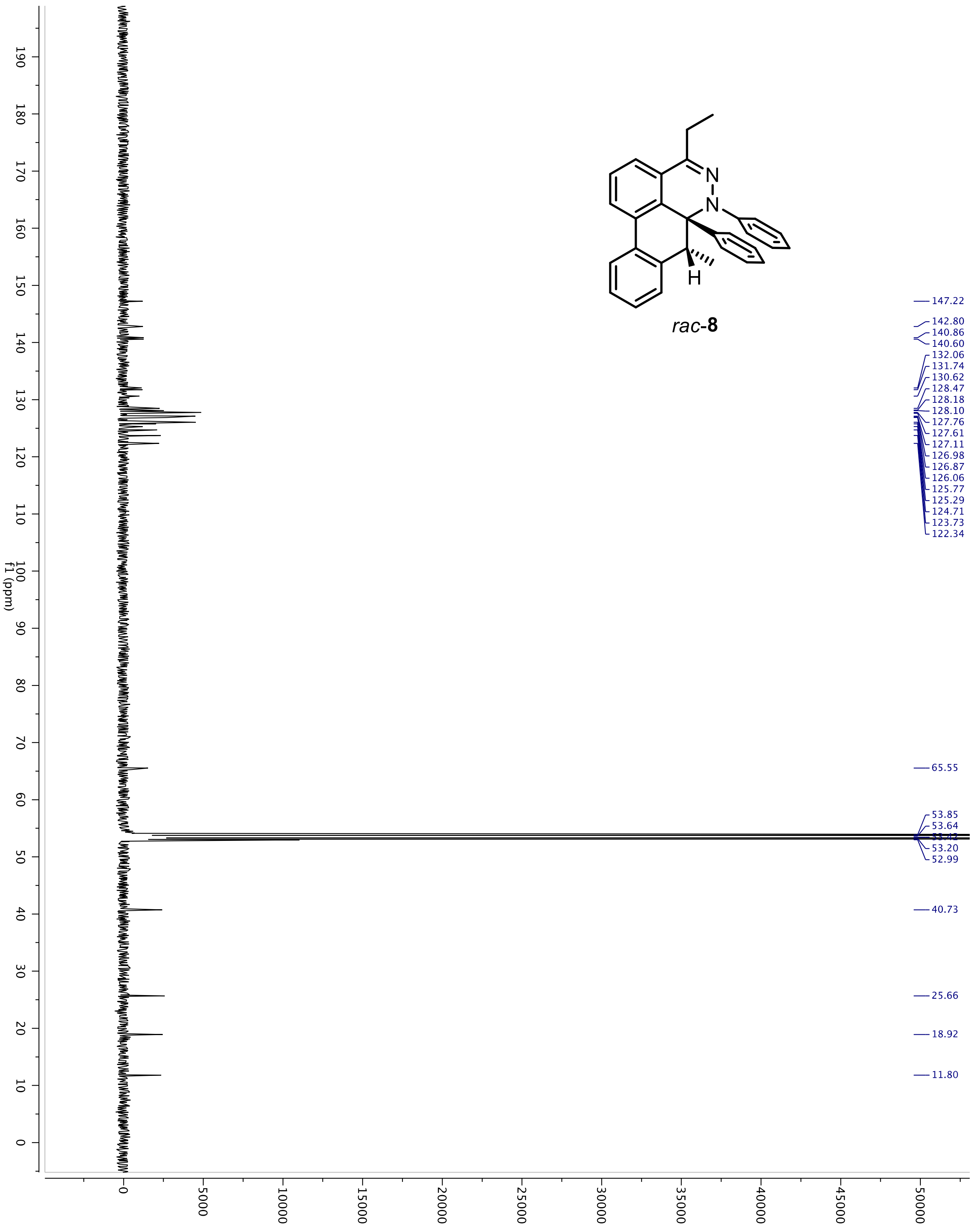

HMBC spectrum of *rac*-**8** in CD_2Cl_2.

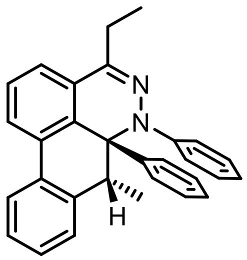

rac-**8**

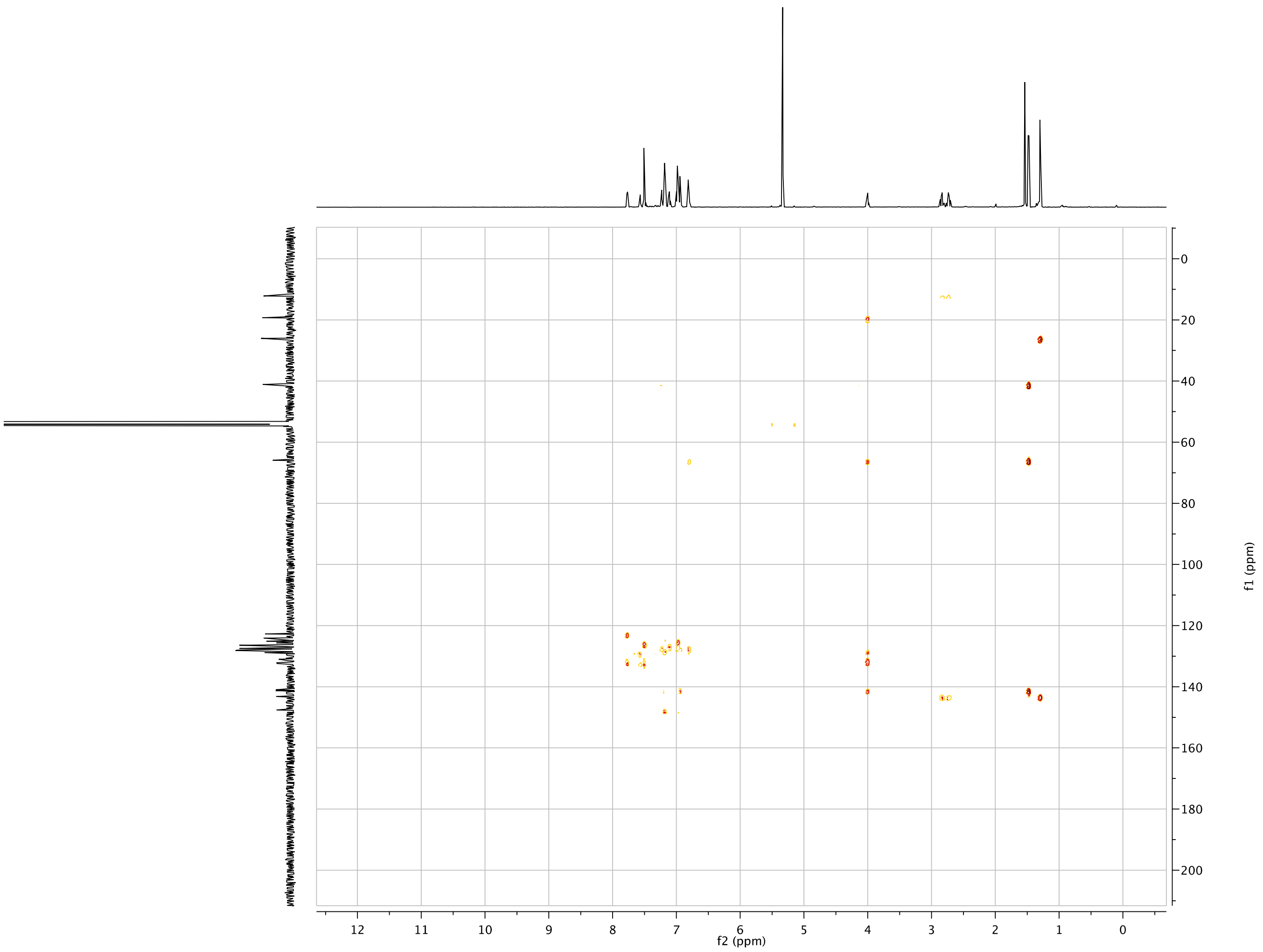

Expanded partial HMBC spectrum of *rac*-**8** in CD_2Cl_2.

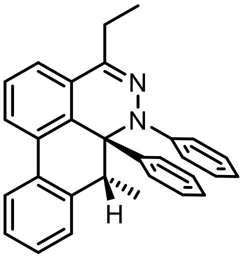

rac-**8**

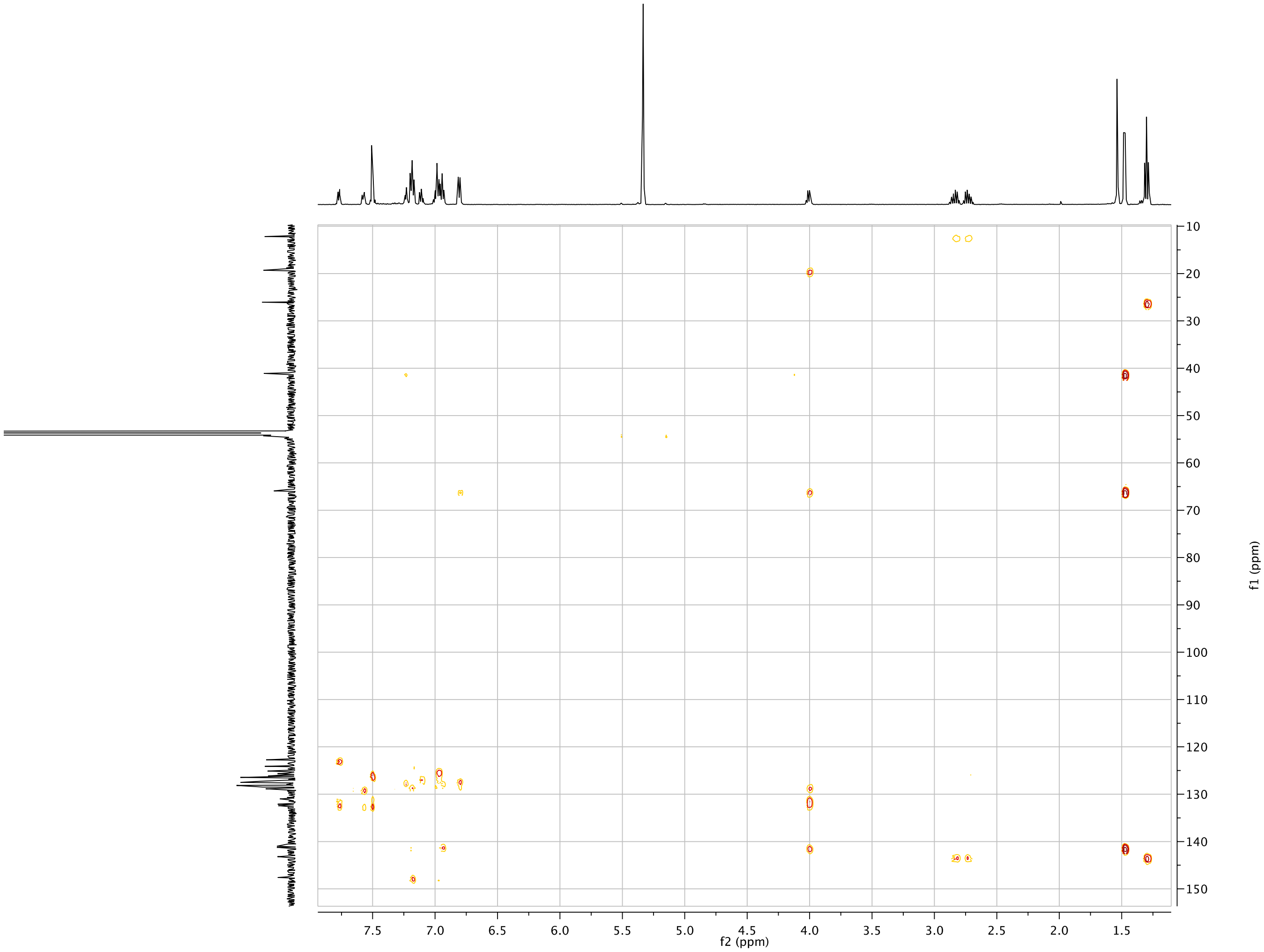

NOESY spectrum of *rac*-**8** in CD_2Cl_2.

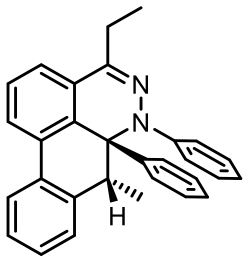

rac-**8**

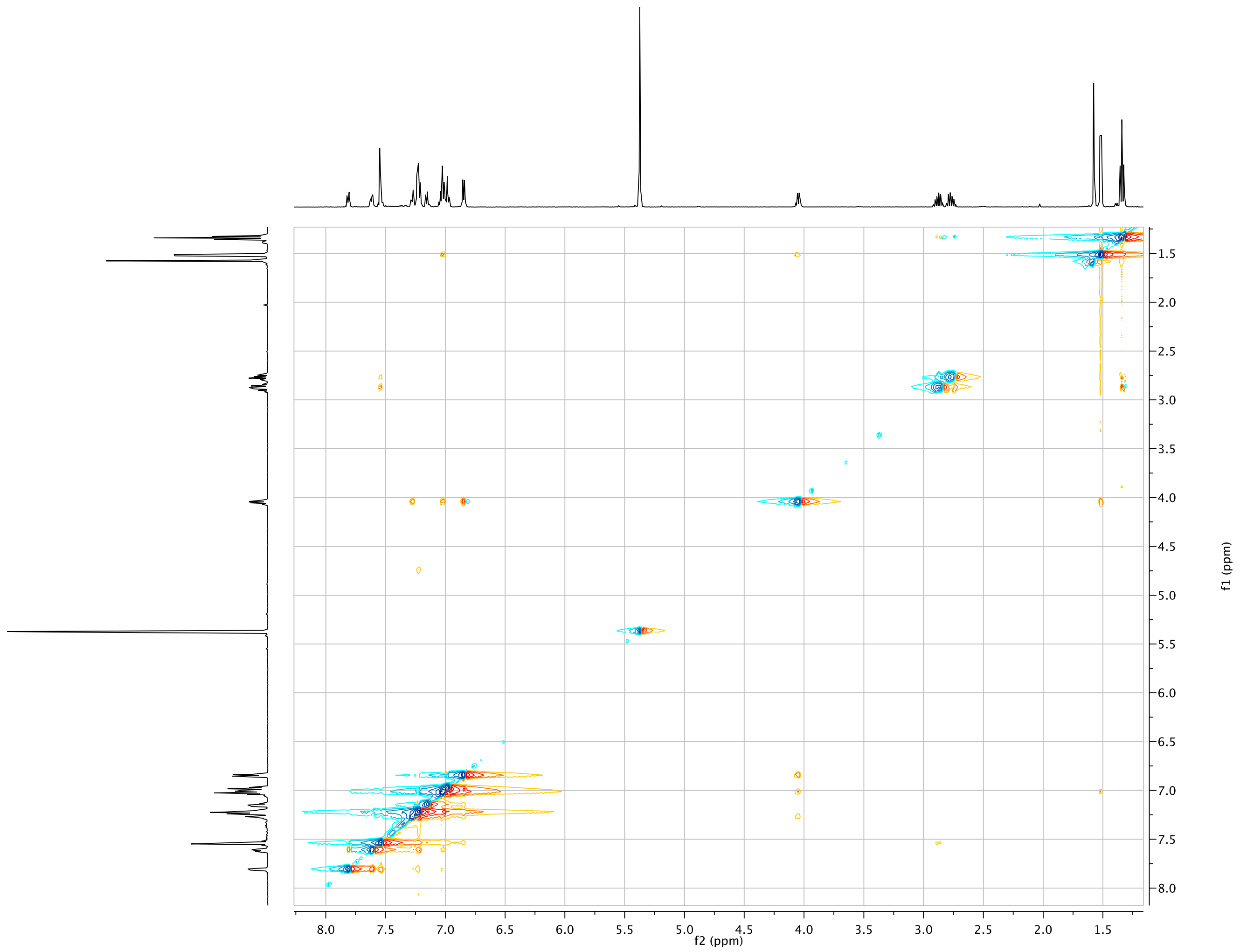

^{1}H NMR spectrum of *rac*-**9a** in CD_2Cl_2 (500 MHz).

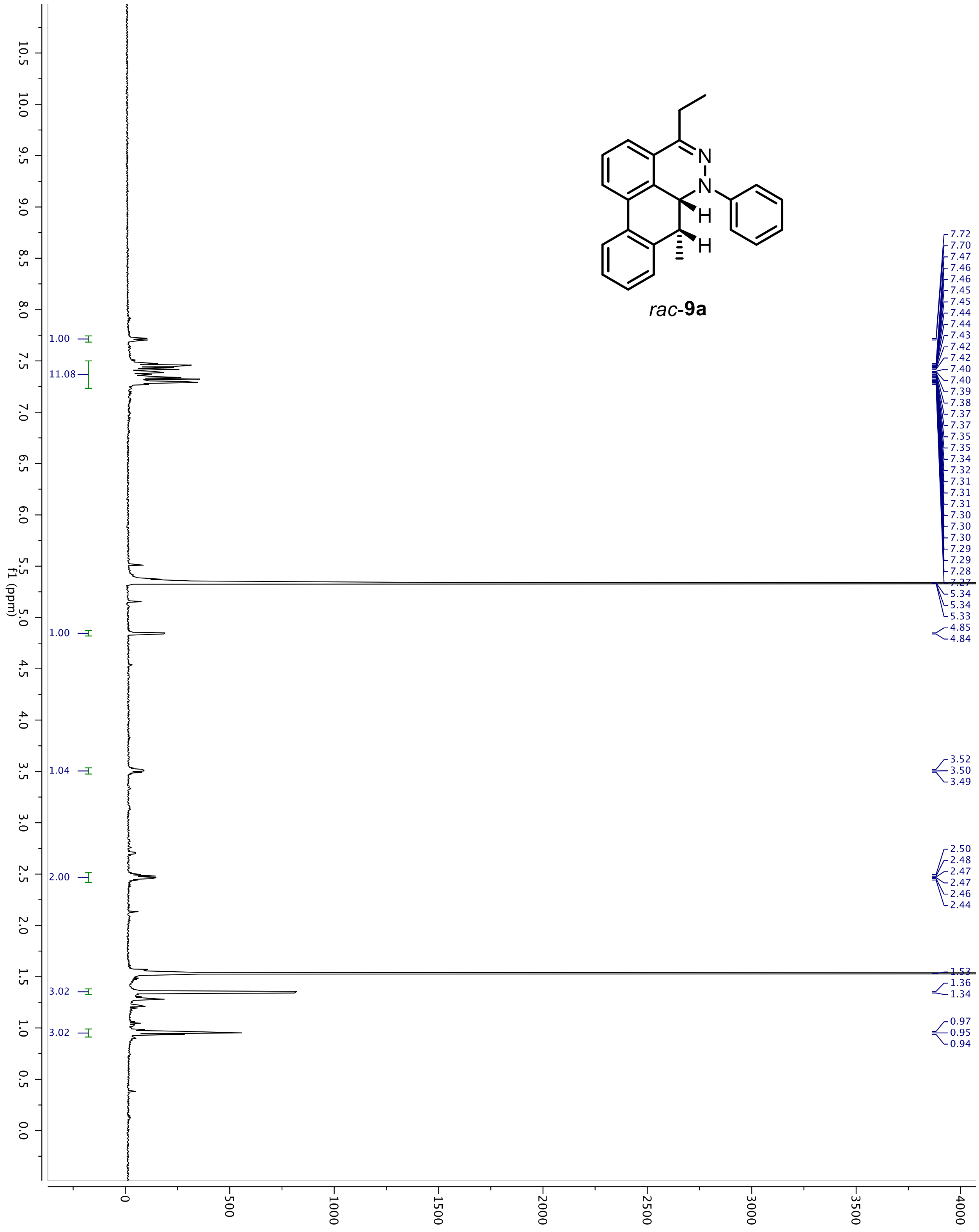

^{1}H NMR spectrum of **24** in CD_2Cl_2 (500 MHz).

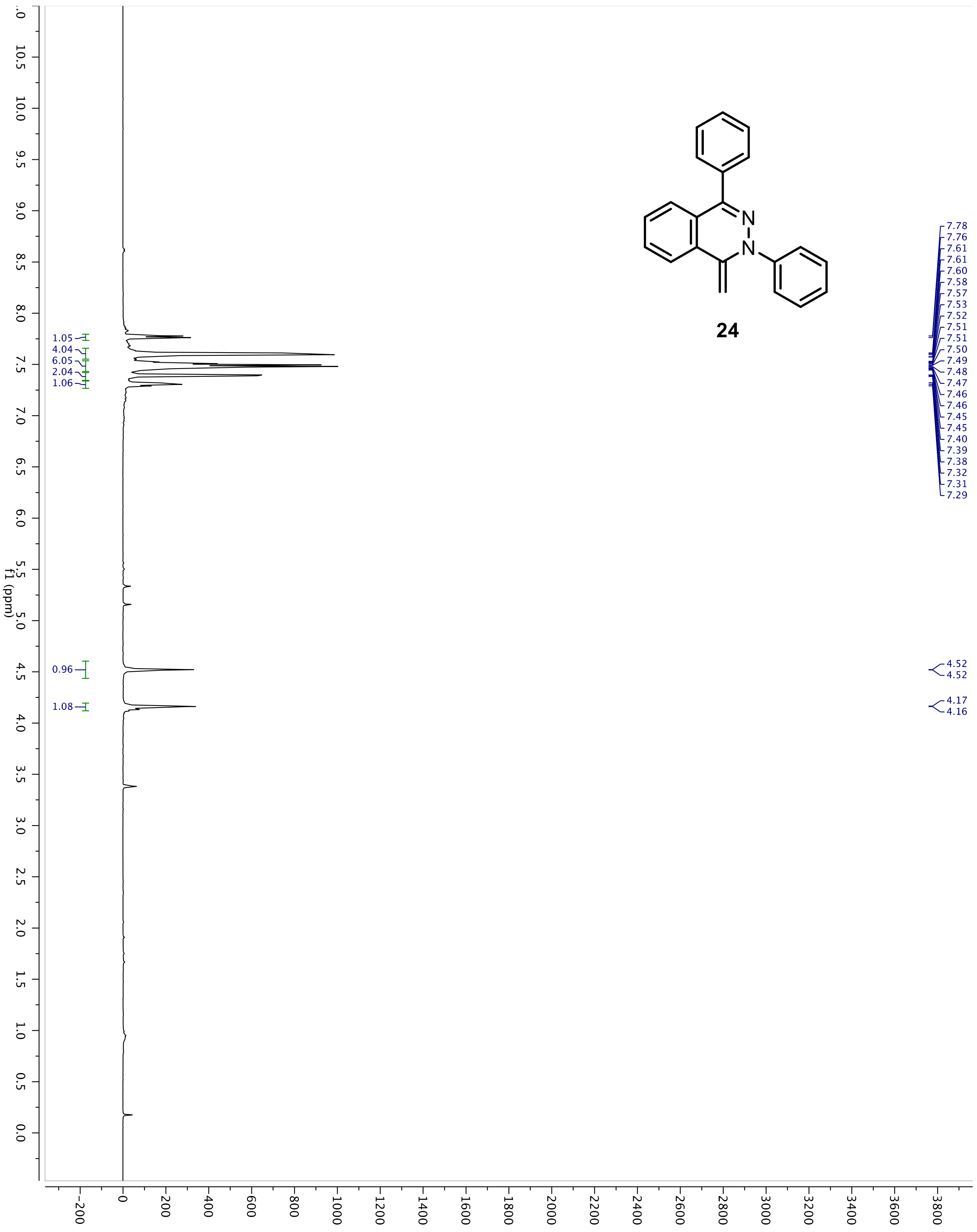

^{13}C NMR spectrum of **24** in CD_2Cl_2 (125 MHz).

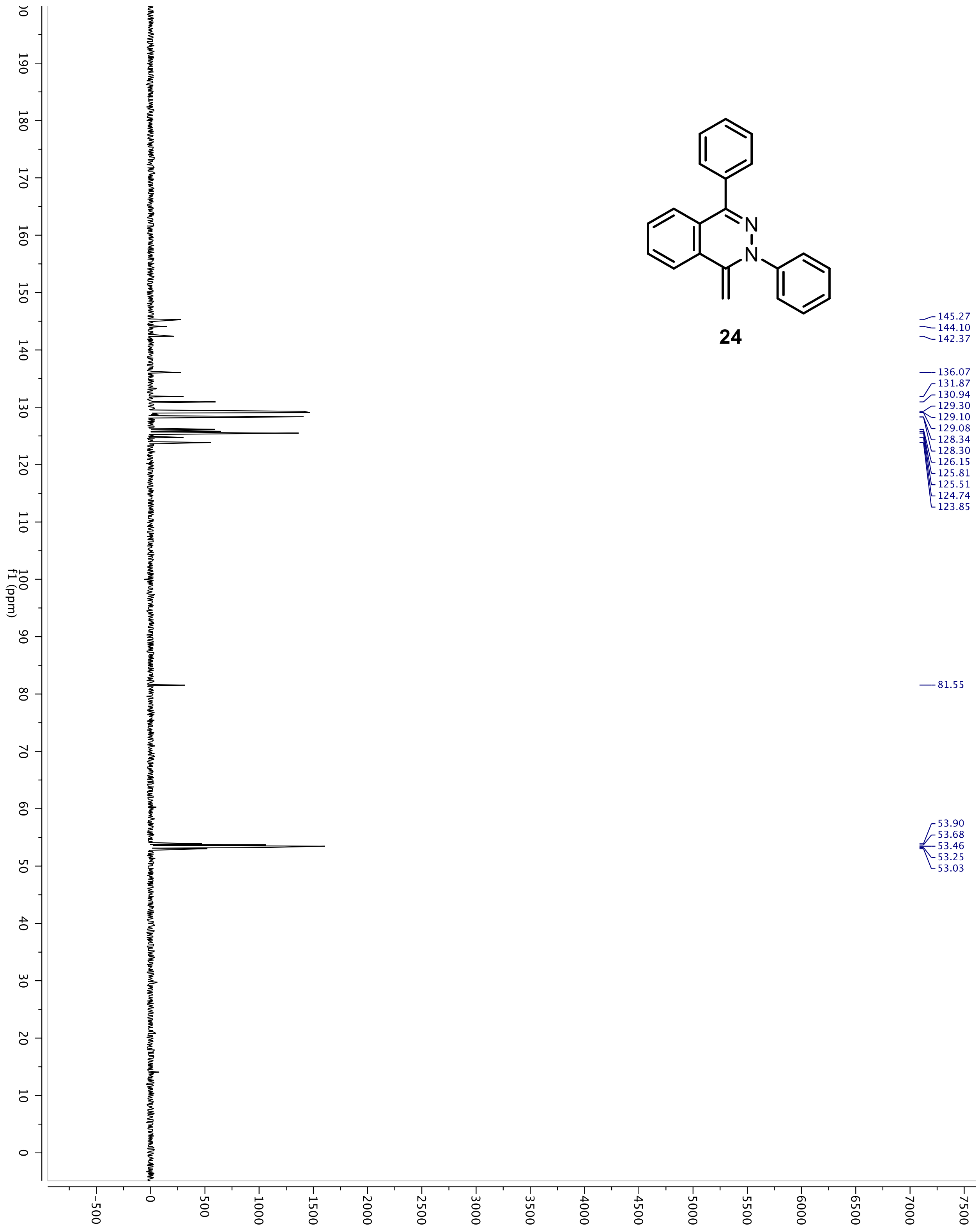

Computational Data

Computed Energies

Structure	ZPE	ΔE	ΔH	ΔG	E_{solv}	H	G_{solv}	Imaginary Frequency (cm-1)
**2**	0.076227	0.080659	0.081603	0.049546	-230.877102	-230.723618	-230.827556	-
**11**	0.107602	0.113636	0.114580	0.077197	-374.922362	-374.694977	-374.845165	-
**TS1**	0.185762	0.198250	0.199194	0.145214	-605.800246	-605.419125	-605.655032	-114.86
**12**	0.188248	0.200215	0.201160	0.149118	-605.828872	-605.439895	-605.679754	-
**TS2**	0.184896	0.197873	0.198817	0.142769	-605.800753	-605.422284	-605.655032	-141.00
**13**	0.191031	0.201717	0.202661	0.156295	-605.937510	-605.562791	-605.781215	-
**TS3**	0.189498	0.200215	0.201159	0.154054	-605.932028	-605.558477	-605.777974	-428.21
**14**	0.181135	0.190870	0.191814	0.146441	-496.505075	-496.166677	-496.358634	-
**N_2**	0.005767	0.008127	0.009072	-0.012671	-109.516789	-109.478340	-109.529460	-
**TS4**	0.258818	0.273881	0.274825	0.214339	-727.385224	-726.895951	-727.170885	-70.37
**15**	0.261402	0.275886	0.276831	0.220036	-727.511696	-726.919698	-727.207521	-
**TS5**	0.258317	0.273282	0.274226	0.215628	-727.378201	-726.889727	-727.162573	-211.97
**16**	0.263812	0.277052	0.277996	0.225488	-727.427557	-727.027638	-727.286208	-
**TS6**	0.261823	0.275220	0.276164	0.223435	-727.502725	-727.021986	-727.279290	-364.47
**17**	0.252987	0.265843	0.266788	0.213694	-618.052072	-617.611343	-617.838378	-
**TS11**	0.281923	0.297810	0.298754	0.238883	-803.864329	-803.298617	-803.625446	-1017.19
**21**	0.263058	0.277159	0.278103	0.221815	-727.497391	-727.012403	-727.283760	-
**H_2O**	0.021540	0.024375	0.025319	0.003885	-76.429020	-76.348059	-76.425135	-
**27**	0.343182	0.362290	0.363234	0.294053	-958.405216	-957.785291	-958.111163	-
**28**	0.345476	0.363778	0.364722	0.298129	958.489900	-958.491403	-958.193274	-
**29**	0.346719	0.364603	0.365547	0.300826	-958.509734	-957.874732	-958.208908	-

Computational Method Benchmarking

Representative transition state structures, as well as the starting materials leading to them, have been calcu lated at the M06-2X/6-311++G(d,p)//B3LYP/6-31G(d) level of theory for comparison. The resulting computed free e nergies of activation are tabulated below in units of kcal/mol.

	M06-2X/6-311++G(d,p)//M06-2X/6-31G(d)	M06-2X/6-311++G(d,p)//B3LYP/6-31G(d)
**TS1**	11.1	13.9
**TS2**	9.2	13.1
**TS3**	14.8	15.6
**TS4**	9.6	9.1

Cartesian Coordinates of Optimized Structures

2

C	0.00000000	0.70249400	1.05307800
C	0.00000000	1.46048400	-0.13268600
C	0.00000000	0.62260200	-1.23250200
C	0.00000000	-0.62260200	-1.23250200
C	0.00000000	-1.46048400	-0.13268600
C	0.00000000	-0.70249400	1.05307800
H	0.00000000	1.22596100	2.00545400
H	0.00000000	2.54455500	-0.13280100
H	0.00000000	-2.54455500	-0.13280100
H	0.00000000	-1.22596100	2.00545400

11

N	0.65298300	-1.18101500	-0.00001500
C	1.28142200	0.00678900	-0.00001600
C	-1.28142200	0.00678900	0.00001300
N	-0.65298300	-1.18101500	0.00000100
N	-0.65751200	1.18758100	0.00001300
N	0.65751200	1.18758100	-0.00001900
C	2.77747400	-0.00569800	0.00002200
H	3.14810600	-0.53361900	0.88276200
H	3.14813500	-0.53589100	-0.88133100
C	-2.77747400	-0.00569800	-0.00000600
H	-3.14811700	-0.53369800	-0.88269400
H	-3.15450900	1.01700000	0.00115100
H	-3.14812400	-0.53581300	0.88139900
H	3.15450900	1.01700000	-0.00121500

12

N	1.09071600	-0.97146200	0.01218500
C	2.39818400	-0.80478700	0.14852000
C	0.95191300	1.34449400	-0.19036800
N	0.35218800	0.12045200	-0.09309200
N	2.26921400	1.44961000	-0.01333600
N	3.00749300	0.39026900	0.20682300
C	3.25053300	-2.02712100	0.26815000
H	3.84108300	-1.97362200	1.18598900
H	2.62695500	-2.92064700	0.28010000
C	0.21414600	2.58708700	-0.52935000
H	-0.48430800	2.40347300	-1.34994800
H	0.95512400	3.34146700	-0.79870200
H	-0.40466900	2.88195300	0.32234900
H	3.94766600	-2.07587200	-0.57257200
C	-2.93169400	-1.48706500	-0.50064800
C	-1.57337900	-1.28091700	-0.60660100
C	-1.08529400	-0.07797100	-0.05564300
C	-1.78559700	0.91738400	0.61132000
C	-3.15179800	0.58422600	0.76223100
C	-3.72141900	-0.54515200	0.18893400
H	-3.38594400	-2.37394900	-0.93086200

H	-0.92320300	-2.01015400	-1.07876500
H	-3.80920200	1.25738900	1.31360000
H	-4.79435100	-0.71319600	0.26146400

13

C	0.00000000	1.23972800	-0.86426300
C	0.00000000	-1.23972800	-0.86426300
N	1.19177700	0.61209700	-1.55433500
N	1.19177700	-0.61209700	-1.55433500
C	0.00000000	-2.73202400	-1.05716000
H	-0.89256200	-3.17041000	-0.60394000
H	0.00000000	-2.96237000	-2.12457400
H	0.89256200	-3.17041000	-0.60394000
C	0.00000000	2.73202400	-1.05716000
H	0.00000000	2.96237000	-2.12457400
H	-0.89256200	3.17041000	-0.60394000
H	0.89256200	3.17041000	-0.60394000
C	0.00000000	0.69846300	2.91992800
C	0.00000000	1.40988000	1.72272200
C	0.00000000	0.69556900	0.53017600
C	0.00000000	-0.69556900	0.53017600
C	0.00000000	-1.40988000	1.72272200
C	0.00000000	-0.69846300	2.91992800
H	0.00000000	1.23586800	3.86287300
H	0.00000000	2.49571600	1.72185800
H	0.00000000	-2.49571600	1.72185800
H	0.00000000	-1.23586800	3.86287300
N	-1.19177700	0.61209700	-1.55433500
N	-1.19177700	-0.61209700	-1.55433500

14

C	2.67090900	-0.70571100	0.00015800
C	1.48497400	-1.40254700	0.00027400
C	0.25653600	-0.70377700	0.00014000
C	0.25652100	0.70378300	-0.00012500
C	1.48494300	1.40258000	-0.00023800
C	2.67089300	0.70577000	-0.00009900
H	3.61515500	-1.24128200	0.00026700
H	1.48700200	-2.48789800	0.00047700
C	-1.02611900	-1.34868600	0.00025300
C	-1.02615000	1.34866400	-0.00026300
H	1.48694800	2.48793100	-0.00044200
H	3.61512800	1.24136200	-0.00019100
N	-2.14965900	-0.67921900	0.00010500
N	-2.14967500	0.67917100	-0.00015400
C	-1.14154800	2.84744700	-0.00052500
H	-0.66251700	3.28169400	0.88360200
H	-0.66255300	3.28137200	-0.88482800
H	-2.19792900	3.11682300	-0.00055400
C	-1.14148100	-2.84747200	0.00048600
H	-0.66241600	-3.28169400	-0.88363500
H	-0.66250000	-3.28139800	0.88479600
H	-2.19785600	-3.11687400	0.00048200

15

C	-4.36661200	-0.24922300	-0.21614600
C	-3.49005400	0.81222900	-0.22529200
C	-2.10938900	0.58196400	-0.06265300
C	-1.62536900	-0.72952500	0.11574100
C	-2.54317800	-1.80532000	0.11918400
C	-3.88759300	-1.56367100	-0.04871800
H	-5.43040000	-0.07624000	-0.34304600
H	-3.85501900	1.82514400	-0.35901000
C	-1.13539500	1.63664400	-0.05519900
C	-0.21134400	-0.92965100	0.26626900
H	-2.18566600	-2.82261800	0.22454300
H	-4.58597300	-2.39413100	-0.05796100
N	0.14050000	1.40172200	0.02057400
N	0.58329800	0.12575300	0.12606300
C	0.33086100	-2.27304100	0.64379500
H	-0.38285000	-2.77275300	1.30609900
H	0.52618600	-2.87219000	-0.24650500
H	1.29303300	-2.15936100	1.14165800
C	-1.53510800	3.07990500	-0.16078200
H	-2.05991200	3.26818200	-1.10292700
H	-2.20395700	3.35984900	0.65913000
H	-0.64100100	3.70191800	-0.12476900
C	2.04156000	-0.00991700	0.00031200
C	2.80100600	0.97673000	0.65013300
C	2.47238900	-1.07172700	-0.79615200
C	4.17398800	0.89899200	0.50825900
H	2.33790500	1.76415500	1.23845500
C	3.88875000	-1.07017600	-0.88220800
C	4.71671000	-0.13417500	-0.27133000
H	4.81818700	1.62610400	0.99361100
H	4.37816900	-1.84708700	-1.47468400
H	5.79737600	-0.19353300	-0.39232900

16

C	3.33967900	-0.69640600	-1.15843300
C	2.27993900	-1.40334800	-0.58967000
C	1.21581700	-0.69867800	-0.04335800
C	1.21585800	0.69867500	-0.04334400
C	2.28002900	1.40327400	-0.58965600
C	3.33972500	0.69626800	-1.15842300
C	0.00002700	-1.28091500	0.63982500
C	-0.00002900	1.28101000	0.63962000
C	-1.21581100	0.69867600	-0.04347400
C	-1.21586400	-0.69867700	-0.04323000
C	-2.28003900	-1.40335500	-0.58943100
H	-2.28787500	-2.48933300	-0.57954400
C	-3.33972700	-0.69643200	-1.15831700
C	-3.33967500	0.69624200	-1.15854500
C	-2.27992800	1.40326700	-0.58989900
H	4.17093800	-1.23646400	-1.60098600
H	2.28773400	-2.48932700	-0.57989200
H	2.28785800	2.48925400	-0.57993900
H	4.17100800	1.23627600	-1.60099200

H	-4.17101200	-1.23650500	-1.60080400
H	-4.17093100	1.23623600	-1.60118200
H	-2.28771600	2.48924800	-0.58029100
C	0.00005200	-2.77875900	0.84121200
H	-0.00000700	-3.30241600	-0.11967500
H	0.88534100	-3.07999100	1.40717300
H	-0.88515800	-3.08000100	1.40728900
C	-0.00005200	2.77888200	0.84080600
H	0.00001100	3.30242600	-0.12014400
H	-0.88534200	3.08018600	1.40672800
H	0.88515600	3.08019300	1.40685100
N	-0.00020700	0.60963000	2.04004800
N	0.00020600	-0.60936400	2.04014000

17

C	-3.65723900	-0.69499600	-0.10920500
C	-2.48219100	-1.38135900	-0.04026400
C	-1.21635400	-0.70987400	0.02666800
C	-1.20989600	0.73037200	0.02422500
C	-2.47290100	1.40717900	-0.05030600
C	-3.65172100	0.72667300	-0.11524800
C	-0.00922500	-1.43162000	0.07856300
C	0.00922500	1.43162000	0.07856400
C	1.21635500	0.70987400	0.02666900
C	1.20989700	-0.73037200	0.02422600
C	2.47290100	-1.40717900	-0.05030600
H	2.49393200	-2.48965400	-0.08585600
C	3.65172200	-0.72667200	-0.11524900
C	3.65723900	0.69499600	-0.10920600
C	2.48219200	1.38135900	-0.04026500
H	-4.59925500	-1.23123800	-0.17059200
H	-2.50399600	-2.46460300	-0.06410300
H	-2.49393100	2.48965500	-0.08585400
H	-4.58979000	1.26911600	-0.18234600
H	4.58979100	-1.26911600	-0.18234800
H	4.59925600	1.23123800	-0.17059400
H	2.50399700	2.46460300	-0.06410400
C	0.04084300	2.93942900	0.16941100
H	0.85136500	3.27393800	0.82030000
H	0.19078200	3.40474000	-0.81234300
H	-0.87923100	3.34177700	0.59187700
C	-0.04084600	-2.93942900	0.16941100
H	-0.85137100	-3.27393600	0.82029700
H	-0.19078400	-3.40474000	-0.81234400
H	0.87922600	-3.34178000	0.59187900

21

C	-4.39477000	-0.28261600	-0.28671300
C	-3.50355000	0.77843100	-0.22812500
C	-2.13796400	0.54475300	-0.02562300
C	-1.67049900	-0.76842200	0.13315800
C	-2.57418500	-1.83102800	0.04776700
C	-3.92585800	-1.59105100	-0.15740000
H	-5.45205900	-0.09528100	-0.44525200

H	-3.86375300	1.79573300	-0.34486300
C	-1.15059000	1.62515100	-0.01367000
C	-0.23389600	-0.97457000	0.41552300
H	-2.21190800	-2.85088300	0.12345900
H	-4.61701800	-2.42543100	-0.22367200
N	0.11566300	1.39565200	-0.01831500
N	0.57448700	0.11668700	0.02453500
C	0.25085500	-2.08150400	1.00079900
C	-1.58053900	3.06272000	-0.07749300
H	-2.13839700	3.26869100	-0.99801900
H	-2.22957800	3.31925600	0.76692100
H	-0.69711300	3.70158500	-0.05470600
C	1.99226900	0.01290600	-0.04313000
C	2.79647400	0.98405500	0.55235100
C	2.57871600	-1.04064800	-0.74860600
C	4.18087300	0.89506100	0.44516900
H	2.32456300	1.80580400	1.07803600
C	3.96232100	-1.12584100	-0.84061300
H	1.94497600	-1.78449500	-1.22067100
C	4.77087400	-0.15988200	-0.24464800
H	4.80062300	1.65535100	0.91089400
H	4.41063800	-1.94666800	-1.39213800
H	5.85132400	-0.22802300	-0.32188200
H	1.30411900	-2.21086500	1.20958800
H	-0.42065100	-2.87622100	1.29628400

27

C	-4.58996300	-0.76632500	-0.39688000
C	-3.69402300	-1.39858500	0.45732000
C	-2.32005500	-1.21045600	0.31658700
C	-1.80018700	-0.37556600	-0.71489100
C	-2.73685100	0.28821300	-1.55113700
C	-4.09375700	0.08420000	-1.39281100
H	-5.65775500	-0.91866100	-0.28452800
H	-4.06659700	-2.03677100	1.25292400
C	-1.35357700	-1.78016700	1.24842300
C	-0.39930500	-0.23624300	-0.84004600
H	-4.78584900	0.59423800	-2.05692700
N	-0.07721900	-1.67337900	1.09766500
N	0.40271900	-0.99031800	0.01699300
C	-1.81306700	-2.54189400	2.45864100
H	-2.45552200	-1.91968500	3.09043400
H	-2.39159900	-3.42495300	2.16622200
H	-0.94448800	-2.86261500	3.03425500
C	1.78992200	-1.19946900	-0.20698500
C	2.67583500	-1.16448800	0.87275600
C	2.26163700	-1.47152400	-1.49182500
C	4.02938100	-1.38370900	0.65698800
H	2.28271400	-0.96622900	1.86382200
C	3.62294300	-1.67649800	-1.69810800
H	1.55523700	-1.54267800	-2.31394500
C	4.51142000	-1.63275600	-0.62834200
H	4.71536500	-1.35130300	1.49784000
H	3.98466800	-1.88840400	-2.69962300
H	5.57156000	-1.79687700	-0.79214400

C	1.37040800	1.97653300	0.49945800
C	1.59860900	2.91796400	1.47325700
C	0.43615300	2.06895600	-0.51074600
C	0.79833800	4.06687800	1.44598300
H	2.36140800	2.78285200	2.23435100
C	-0.34823800	3.22863700	-0.51612200
C	-0.16778700	4.21504700	0.45379500
H	0.93232800	4.83865500	2.19839700
H	-1.10471200	3.35751600	-1.28758800
H	-0.78737000	5.10617500	0.43283500
C	0.23841500	0.94789800	-1.51908600
H	1.20345500	0.69162200	-1.96182500
H	-0.39825200	1.29757100	-2.33378000
H	-2.39059400	0.94554900	-2.34070800

28

C	-4.57237800	-0.26567000	-0.59099300
C	-3.76949000	-1.11266500	0.16267500
C	-2.37891500	-0.97120000	0.14222000
C	-1.78894500	0.02141900	-0.65190900
C	-2.60254600	0.88427100	-1.38164100
C	-3.98683500	0.73779900	-1.35863900
H	-5.65151300	-0.38103500	-0.57259100
H	-4.22334400	-1.88843900	0.77198900
C	-1.48774200	-1.83431500	0.93031400
C	-0.28534900	0.17713500	-0.54856800
H	-4.60841500	1.41484100	-1.93639400
N	-0.22431500	-1.89986200	0.70662400
N	0.30363600	-1.15137500	-0.32396300
C	-2.04156800	-2.73003100	2.00211500
H	-2.61434900	-2.15914400	2.74104300
H	-2.71343200	-3.48484000	1.57723500
H	-1.21739900	-3.24121700	2.50086400
C	1.73933600	-1.21781300	-0.30521600
C	2.45933100	-0.93998300	0.85646900
C	2.41140300	-1.58333100	-1.46937600
C	3.84813300	-1.01427300	0.84480500
H	1.91863500	-0.67987800	1.76090000
C	3.80239100	-1.64635800	-1.48126300
H	1.82858100	-1.82629900	-2.35303100
C	4.52249200	-1.36077600	-0.32444800
H	4.40601900	-0.79731900	1.75060600
H	4.32191900	-1.93150500	-2.39095000
H	5.60672600	-1.41623800	-0.33100200
C	0.12033800	1.31414600	0.40706900
C	0.07082000	1.74692200	1.72239800
C	0.65224300	2.08150000	-0.61867000
C	0.60126600	3.01880100	1.96099400
H	-0.35114400	1.14972200	2.52578500
C	1.16781700	3.34836400	-0.39721700
C	1.13209200	3.79918700	0.92681500
H	0.59862100	3.41842800	2.97093400
H	1.58468800	3.96710000	-1.18624200
H	1.52727200	4.78279400	1.16394900
C	0.40569600	1.02684800	-1.68127400

H	1.31535000	0.56601000	-2.06955600
H	-0.23664600	1.29342800	-2.52365300
H	-2.16436100	1.69237500	-1.95847400

29

C	3.25940600	2.50766800	-0.17567400
C	1.93892300	3.08266400	-0.37187700
C	0.84426700	2.32009600	-0.12058500
C	0.97063300	0.96624500	0.36689400
C	2.31987400	0.38619500	0.76498300
C	3.45480600	1.27792600	0.32514900
H	4.12246900	3.11495200	-0.43578800
H	1.84706900	4.10421800	-0.72234000
C	-0.54067900	2.76516900	-0.28063800
C	-0.13047500	0.19080600	0.50931900
H	4.46486500	0.91394900	0.49060700
N	-1.56803500	1.99316900	-0.14480400
N	-1.38131800	0.68554600	0.18472100
C	-0.82048500	4.19112700	-0.65048900
H	-0.39071000	4.87214400	0.09207300
H	-0.35997100	4.42852300	-1.61607300
H	-1.89654600	4.35384500	-0.71624600
C	-2.54567900	-0.11876200	0.04044600
C	-3.75992700	0.33708600	0.55340600
C	-2.48474400	-1.32991200	-0.65131900
C	-4.90490500	-0.43345500	0.39370300
H	-3.78868000	1.29515900	1.06051100
C	-3.63378900	-2.10252300	-0.78882200
H	-1.54392900	-1.65022700	-1.09197100
C	-4.84595900	-1.65928500	-0.26708200
H	-5.84860300	-0.07631600	0.79414200
H	-3.58201000	-3.04465400	-1.32572700
H	-5.74228400	-2.26012900	-0.38310200
C	2.39366300	-1.06306800	0.28613900
C	3.49866700	-1.63306800	-0.33693300
C	1.25017600	-1.84411400	0.50091800
C	3.47473100	-2.97234100	-0.72707300
H	4.37921500	-1.03169100	-0.53958500
C	1.22582800	-3.17710400	0.10600200
C	2.34247400	-3.74707400	-0.50350500
H	4.34260300	-3.40569600	-1.21487200
H	0.32969600	-3.77020700	0.27429000
H	2.32171500	-4.78801400	-0.81116100
C	0.05839400	-1.16743100	1.14126800
H	-0.83037500	-1.79547400	1.10073400
H	2.35289700	0.32076600	1.87223400
H	0.27684300	-0.99323200	2.20748400

H_2O

O	0.00000000	0.00000000	0.11846300
H	0.00000000	-0.76239800	-0.47385000
H	0.00000000	0.76239800	-0.47385000

N_2

N	0.00000000	0.00000000	0.54938200
N	0.00000000	0.00000000	-0.54938200

TS1

N	-1.23575200	-0.95435500	0.32840900
C	-2.47461500	-0.89899200	-0.15964300
C	-1.24793000	1.33346900	0.26301800
N	-0.62090400	0.18350300	0.53927900
N	-2.49303100	1.37378600	-0.24403400
N	-3.11257100	0.24675600	-0.46124800
C	-3.20756800	-2.18051700	-0.39549100
H	-3.51463500	-2.24571600	-1.44242000
H	-2.56687200	-3.02614300	-0.14594100
C	-0.53777800	2.61710700	0.50818700
H	-0.09926300	2.62369000	1.50879800
H	-1.23576600	3.44565400	0.38966000
H	0.29459700	2.70112400	-0.20238400
H	-4.11252700	-2.21192400	0.21718100
C	3.18737000	-1.41975000	0.39525600
C	1.84166600	-1.18897300	0.71296300
C	1.45142800	0.00956400	0.17249600
C	1.99887900	0.92560700	-0.52328800
C	3.34637000	0.68522200	-0.84717800
C	3.92139000	-0.49822100	-0.36973800
H	3.66456200	-2.32978700	0.74751900
H	1.22408600	-1.87173000	1.28451600
H	3.94338900	1.37528500	-1.44048900
H	4.96295800	-0.71538900	-0.59278100

TS2

C	-1.50560100	1.26913200	-0.00012100
C	-1.50646300	-1.26862100	0.00074700
N	-1.69995900	0.64973900	-1.18226400
N	-1.70020100	-0.64985500	-1.18183800
C	-1.35317700	-2.75682100	0.00139300
H	-0.80694000	-3.07244000	0.89194900
H	-2.33776300	-3.23519900	0.01295300
H	-0.82573900	-3.07506800	-0.89945300
C	-1.35133900	2.75723500	-0.00075200
H	-2.33566900	3.23628300	-0.00105600
H	-0.81431400	3.07416000	0.89490700
H	-0.81419900	3.07345200	-0.89659500
C	3.30030600	0.70164200	0.00154200
C	2.11356600	1.45685900	0.00076800
C	1.00865800	0.62694000	-0.00167200
C	1.00857200	-0.62756400	-0.00300700
C	2.11337100	-1.45763100	-0.00246400
C	3.30020900	-0.70256800	-0.00002600
H	4.25219900	1.22556200	0.00348800
H	2.11201400	2.54144900	0.00201000
H	2.11168400	-2.54222000	-0.00369800
H	4.25203500	-1.22661900	0.00071900

N	-1.69689600	0.65045400	1.18300700
N	-1.69750400	-0.64904900	1.18342700

TS3

C	-2.91873900	-0.70072500	0.02574600
C	-1.72631000	-1.40816000	-0.01756100
C	-0.52515600	-0.69771800	-0.07689900
C	-0.52515600	0.69771800	-0.07689900
C	-1.72631000	1.40816000	-0.01756100
C	-2.91873900	0.70072500	0.02574600
H	-3.86143000	-1.23733700	0.06354300
H	-1.72551700	-2.49401100	-0.01291500
H	-1.72551700	2.49401100	-0.01291500
H	-3.86143000	1.23733700	0.06354300
C	0.83269300	1.26594000	-0.12694800
C	0.83269300	-1.26594000	-0.12694800
N	1.51718000	0.58579100	1.32539500
N	1.51718000	-0.58579100	1.32539500
N	1.64540800	0.62980200	-1.10706300
N	1.64540800	-0.62980200	-1.10706300
C	1.03436000	2.75127400	-0.05037400
C	1.03436000	-2.75127400	-0.05037400
H	2.10231400	2.97553400	-0.07600300
H	0.55631800	3.24265300	-0.90268100
H	0.60910700	3.14534300	0.87594400
H	2.10231400	-2.97553300	-0.07600300
H	0.60910700	-3.14534300	0.87594400
H	0.55631800	-3.24265200	-0.90268100

TS4

C	-4.62891500	-0.06695900	-0.00035400
C	-3.68431900	0.93312000	-0.00016300
C	-2.31098400	0.60245000	0.00002500
C	-1.91933100	-0.74913400	0.00002100
C	-2.90340100	-1.76250300	-0.00017600
C	-4.23641000	-1.42223200	-0.00036400
H	-5.68484400	0.18439900	-0.00050100
H	-3.98889200	1.97478300	-0.00015800
C	-1.26075100	1.58129800	0.00022000
C	-0.50737800	-1.02497000	0.00021700
H	-2.60227300	-2.80489000	-0.00018100
H	-4.99447000	-2.19924600	-0.00052700
N	0.00196000	1.24615900	0.00036800
N	0.36817800	-0.05397200	0.00036800
C	0.00916200	-2.43468800	0.00026100
H	-0.35007700	-2.97159900	-0.88412100
H	1.09988000	-2.43038300	0.00045500
H	-0.35037400	-2.97164400	0.88449700
C	-1.56529000	3.05226800	0.00025700
H	-2.14613600	3.33387500	-0.88422900
H	-2.14641100	3.33376900	0.88459600
H	-0.62779300	3.60849400	0.00043600
C	2.63565100	-0.18578200	0.00015900
C	2.99840500	1.13477600	-0.00021900

C	3.22321800	-1.31309800	0.00030000
C	4.40026700	1.21344100	-0.00048700
H	2.33592300	1.99000000	-0.00029800
C	4.62792000	-1.22293400	0.00003000
C	5.19567200	0.05656500	-0.00036400
H	4.87158800	2.19256800	-0.00081700
H	5.27425900	-2.09938400	0.00010600
H	6.27755500	0.16424100	-0.00058800

TS5

C	2.80839600	0.70504200	-1.87637000
C	2.09850200	1.40357900	-0.92441900
C	1.37815200	0.70227400	0.06378600
C	1.37832500	-0.70211900	0.06369400
C	2.09885200	-1.40311800	-0.92459900
C	2.80857300	-0.70428300	-1.87646000
H	3.36965600	1.24077500	-2.63552700
H	2.09720800	2.48909300	-0.92685600
C	0.60893500	1.33331200	1.10356000
C	0.60926900	-1.33348700	1.10338400
H	2.09782800	-2.48863100	-0.92717400
H	3.36997100	-1.23977700	-2.63568400
N	0.21427100	0.66160200	2.18350400
N	0.21442700	-0.66202200	2.18341100
C	0.46102300	-2.82750500	1.16407000
H	0.06153100	-3.22243200	0.22504000
H	1.42929600	-3.30807700	1.34555500
H	-0.21386200	-3.08186000	1.98207600
C	0.46033500	2.82728800	1.16445100
H	1.42850300	3.30807400	1.34592100
H	0.06067400	3.22223300	0.22550000
H	-0.21455300	3.08137300	1.98253800
C	-3.52926500	0.70126400	-1.16702000
C	-2.50558600	1.44876100	-0.55717600
C	-1.55171300	0.63112400	0.01635700
C	-1.55166600	-0.63145600	0.01618800
C	-2.50551600	-1.44895600	-0.55759100
C	-3.52923000	-0.70133100	-1.16722100
H	-4.34792400	1.22667500	-1.65142300
H	-2.50790000	2.53452500	-0.55683500
H	-2.50778700	-2.53472000	-0.55757200
H	-4.34786200	-1.22664200	-1.65177700

TS6

C	-3.48368700	0.70023500	-0.95745000
C	-2.36798800	1.39958400	-0.52659400
C	-1.21928000	0.70333200	-0.12994200
C	-1.21928000	-0.70333200	-0.12994200
C	-2.36798800	-1.39958400	-0.52659400
C	-3.48368700	-0.70023500	-0.95745000
C	0.00000000	1.32686000	0.40469500
C	0.00000000	-1.32686000	0.40469500
C	1.21928000	-0.70333200	-0.12994200
C	1.21928000	0.70333200	-0.12994200

C	2.36798800	1.39958400	-0.52659400
H	2.37528400	2.48548100	-0.51647300
C	3.48368700	0.70023500	-0.95745000
C	3.48368700	-0.70023500	-0.95745000
C	2.36798800	-1.39958400	-0.52659400
H	-4.36374400	1.24078800	-1.29194200
H	-2.37528400	2.48548100	-0.51647300
H	-2.37528400	-2.48548100	-0.51647300
H	-4.36374400	-1.24078800	-1.29194200
H	4.36374400	1.24078800	-1.29194200
H	4.36374400	-1.24078800	-1.29194200
H	2.37528400	-2.48548100	-0.51647300
C	0.00000000	2.81044200	0.65845500
H	0.00000000	3.37206400	-0.28224600
H	-0.88393400	3.09536100	1.23427200
H	0.88393400	3.09536100	1.23427200
C	0.00000000	-2.81044200	0.65845500
H	0.00000000	-3.37206400	-0.28224600
H	0.88393400	-3.09536100	1.23427100
H	-0.88393400	-3.09536100	1.23427100
N	0.00000000	-0.58455200	2.05992300
N	0.00000000	0.58455100	2.05992300

TS11

C	4.47541900	-0.22658200	0.04077200
C	3.62795500	0.83710600	0.26315600
C	2.24156700	0.67739700	0.07857300
C	1.72241200	-0.56053700	-0.34376800
C	2.60565600	-1.64032000	-0.55717100
C	3.95938200	-1.47120100	-0.36353000
H	5.54451200	-0.10782600	0.18506500
H	4.02159600	1.79470200	0.58715300
C	1.29761900	1.73876700	0.31571600
C	0.29443600	-0.69414800	-0.52490800
H	2.21877800	-2.60963700	-0.84603600
H	4.63347600	-2.30709000	-0.51919100
N	0.01785800	1.56038800	0.21306000
N	-0.46061500	0.34548200	-0.16712500
C	-0.30362200	-1.94145700	-1.00782100
H	0.37313700	-2.47373900	-1.67473500
H	-0.56122800	-2.55875700	-0.05594600
H	-1.26455200	-1.76588100	-1.49244000
C	1.74325600	3.11263100	0.72547200
H	2.27356900	3.07892300	1.68265500
H	2.42046200	3.54164000	-0.01973200
H	0.86925800	3.75560200	0.82865000
C	-1.92736200	0.29556400	-0.13810300
C	-2.58685600	1.35235900	-0.77122400
C	-2.53831000	-0.74560400	0.55296000
C	-3.97245600	1.36924500	-0.73307100
H	-2.02727600	2.13641100	-1.27252300
C	-3.94131900	-0.67299900	0.56191500
C	-4.65141300	0.35018000	-0.05878000
H	-4.51853700	2.16997700	-1.22209300
H	-4.49457300	-1.45455300	1.07993200

H	-5.73800200	0.36324100	-0.02364400
O	-1.29239000	-2.88742800	1.37574100
H	-1.89843700	-1.82488100	1.09186000
H	-0.69195000	-2.66220400	2.10242200

rac-**9a**

C	2.20696400	0.76324500	-0.33410000
C	0.82603000	0.94611800	-0.35275900
C	0.27449200	2.21329900	-0.38958400
C	2.74128700	-0.61422500	-0.16530300
C	1.92431800	-1.57456400	0.43808900
C	0.49591000	-1.21112900	0.81192800
C	-0.03763900	-0.30093700	-0.32032900
N	-1.45794100	0.02696700	-0.16176200
N	-1.95828500	1.32841300	-0.19055300
C	-1.19418800	2.32991100	-0.30282700
C	-2.45495100	-0.96248600	-0.24274600
H	0.11139700	-0.82194900	-1.26366500
H	-0.09384000	-2.11601400	0.84248200
C	0.41677400	-0.52870000	2.18897500
H	0.75732400	-1.21187900	2.95825200
H	1.03793400	0.35617700	2.21765500
H	-0.60637300	-0.24707000	2.40557100
C	-1.78046700	4.33937100	1.11752100
H	-2.25432100	5.31479200	1.11155900
H	-2.29545400	3.71083400	1.83356300
H	-0.75544300	4.45835400	1.44598600
C	-1.85504200	3.69096500	-0.28521100
H	-2.89279600	3.54706700	-0.55160300
H	-1.40828500	4.34049500	-1.02783000
C	1.11611600	3.31742300	-0.46768100
H	0.70819600	4.30516300	-0.50629100
C	2.48478100	3.14686100	-0.47865500
H	3.12928100	4.00150400	-0.52877200
C	3.02969800	1.87895800	-0.39300000
H	4.09302300	1.76390400	-0.34536500
C	4.02651000	-0.96581600	-0.55972300
H	4.64618400	-0.24915800	-1.05983400
C	4.51144100	-2.23994700	-0.33333300
H	5.50378800	-2.49675000	-0.64627900
C	3.71119000	-3.18122600	0.28804600
H	4.08149500	-4.17109400	0.46668800
C	2.42247400	-2.84629200	0.66332100
H	1.79460300	-3.58111000	1.12920000
C	-2.18445600	-2.22808800	-0.75262700
H	-1.21195900	-2.47117500	-1.12343500
C	-3.17560900	-3.19446900	-0.80739900
H	-2.93794200	-4.16063800	-1.20753700
C	-4.45338300	-2.92204000	-0.36610200
H	-5.21802700	-3.67111600	-0.40973100
C	-4.73073900	-1.65756000	0.13039300
H	-5.71874700	-1.42265200	0.47489700
C	-3.75203700	-0.69026400	0.19383400
H	-3.97330300	0.28420700	0.56632800

rac-**9b**

C	2.23216000	0.75706900	-0.42786000
C	0.85975000	0.97079600	-0.33828800
C	0.33338000	2.24726700	-0.36285800
C	2.73745100	-0.61824300	-0.18305900
C	1.97681600	-1.45929400	0.64464800
C	0.62039600	-0.94524400	1.14313300
C	-0.00577000	-0.25475600	-0.11081200
N	-1.42412000	0.09680900	-0.01900500
N	-1.88629800	1.41513800	0.07105600
C	-1.10938500	2.39902800	-0.09591900
C	-2.45545400	-0.83490500	-0.25854300
H	0.79750100	-0.16119100	1.87436100
H	0.13612900	-0.91523600	-0.95659700
C	1.18493000	3.32464100	-0.58548400
H	0.79652000	4.32044600	-0.62367100
C	2.54040000	3.11831600	-0.73843600
H	3.19192100	3.95294500	-0.90330900
C	3.06837100	1.84336800	-0.63335200
H	4.12915400	1.70248100	-0.67741100
C	3.93641200	-1.07044000	-0.71200300
H	4.49745900	-0.43960900	-1.37215900
C	4.40719700	-2.33582800	-0.41118800
H	5.33367300	-2.67586200	-0.82901300
C	3.67441300	-3.15634300	0.42244700
H	4.03136800	-4.13814400	0.66281100
C	2.46570600	-2.71912500	0.94126900
H	1.90594900	-3.37382200	1.57550900
C	-3.76885200	-0.53302800	0.10522400
H	-3.97230500	0.41415100	0.54997200
C	-4.78590900	-1.43531400	-0.11952400
H	-5.78457200	-1.17683600	0.17379800
C	-4.53431700	-2.66181400	-0.71320000
H	-5.32869000	-3.35981600	-0.88475400
C	-3.23967400	-2.96315200	-1.08151400
H	-3.01728300	-3.90175900	-1.55028100
C	-2.20966400	-2.06412600	-0.86118900
H	-1.22399700	-2.33835400	-1.16716000
C	-0.24947900	-2.01974900	1.80826600
H	-1.21254900	-1.60186100	2.06501700
H	-0.41000500	-2.87122700	1.16157600
H	0.22092500	-2.36077800	2.72390700
C	-1.41306500	4.40095400	1.42072700
H	-1.85475500	5.38826100	1.49792700
H	-1.82933400	3.77613100	2.20142300
H	-0.34667000	4.48867300	1.58740800
C	-1.72013900	3.77633700	0.03921600
H	-2.78982100	3.66413700	-0.06882300
H	-1.37172600	4.42515000	-0.75509200
